# Maternal Undernutrition Induces Cell Signalling and Metabolic Dysfunction in Undifferentiated Mouse Embryonic Stem Cells

**DOI:** 10.1007/s12015-022-10490-1

**Published:** 2022-12-15

**Authors:** Pooja Khurana, Andrew Cox, Barira Islam, Judith J. Eckert, Sandrine Willaime-Morawek, Joanna M. Gould, Neil R. Smyth, Patrick C. McHugh, Tom P. Fleming

**Affiliations:** 1 School of Biological Sciences, Mailpoint 840, Level D Lab & Path Block, Southampton General Hospital, University of Southampton, Tremona Road, Southampton, SO16 6YD UK; 2 Centre for Biomarker Research, School of Applied Sciences, University of Huddersfield, Huddersfield, HD1 3DH UK; 3 Faculty of Medicine, Southampton General Hospital, University of Southampton, Southampton, SO16 6YD UK

**Keywords:** Mouse ES cells, Maternal low protein diet, RNAseq, Metabolomics, MAPK pathway, Glucose metabolism, Cell signalling, DOHaD

## Abstract

Peri-conceptional environment can induce permanent changes in embryo phenotype which alter development and associate with later disease susceptibility. Thus, mouse maternal low protein diet (LPD) fed exclusively during preimplantation is sufficient to lead to cardiovascular, metabolic and neurological dysfunction in adult offspring. Embryonic stem cell (ESC) lines were generated from LPD and control NPD C57BL/6 blastocysts and characterised by transcriptomics, metabolomics, bioinformatics and molecular/cellular studies to assess early potential mechanisms in dietary environmental programming. Previously, we showed these lines retain cellular and epigenetic characteristics of LPD and NPD embryos after several passages. Here, three main changes were identified in LPD ESC lines. First, their derivation capacity was reduced but pluripotency marker expression was similar to controls. Second, LPD lines had impaired Mitogen-activated protein kinase (MAPK) pathway with altered gene expression of several regulators (e.g., *Maff*, *Rassf1*, *JunD*), reduced ERK1/2 signalling capacity and poorer cell survival characteristics which may contribute to reduced derivation. Third, LPD lines had impaired glucose metabolism comprising reduced upstream enzyme expression (e.g., *Gpi, Mpi*) and accumulation of metabolites (e.g., glucose-6-P, fructose-6-P) above the phosphofructokinase (PFK) gateway with PFK enzyme activity reduced. ESC lines may therefore permit investigation of peri-conceptional programming mechanisms with reduced need for animal experimentation.

**Graphical Abstract:**

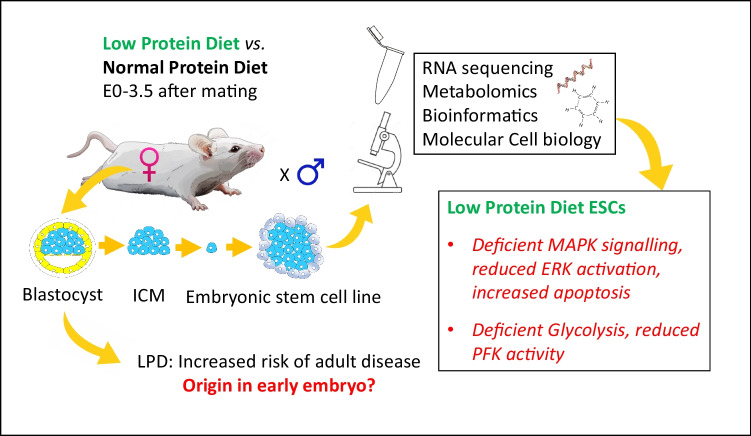

**Supplementary Information:**

The online version contains supplementary material available at 10.1007/s12015-022-10490-1.

## Introduction

The origin of adult chronic non-communicable diseases across the cardiometabolic and neurological spectrum can arise from the prenatal environment according to the Developmental Origins of Health and Disease (DOHaD) hypothesis [1]. In particular, the period around conception is vulnerable to diverse stress conditions including sub-optimal parental nutrition and assisted reproductive treatments (ART) [2–4]. Adverse peri-conceptional environment can alter multiple molecular, cellular and physiological processes in the developmental programme that persist through gestation affecting growth, metabolism and later disease risk, across mammalian species, including humans [2, 5, 6]. Whilst challenging environmental conditions can evoke developmental plasticity to aid survival, altered programming can become maladaptive where conditions later change leading to pathophysiology [1, 2, 4].

We have used a mouse maternal protein restriction model whereby low protein diet (LPD), administered exclusively during preimplantation development with normal nutrition thereafter and postnatally, is sufficient to induce adult offspring cardiovascular, metabolic, structural and neurological dysfunction [7–10]. This transient restriction alters uterine metabolite and amino acid levels sensed directly by the preimplantation embryo through mTOR signalling [11] leading to an array of cellular responses affecting both embryonic [8–10, 12] and extra-embryonic [11, 13–16] lineages. Given the limited size and inaccessibility of the preimplantation embryo, we have used embryonic stem cell (ESC) lines derived from blastocysts to probe effects of maternal LPD in early embryonic programming. This novel approach has shown common molecular and epigenetic changes induced by maternal diet in both early passage ESC lines and embryos *in vivo* [13, 14]. Moreover, ESC lines have been employed for mechanistic analysis of other peri-conceptional conditions associated with DOHaD [17, 18].

Here, we provide a detailed characterisation of undifferentiated LPD and control (normal protein diet, NPD) ESC lines for further evidence of early steps in adverse programming and phenotypic plasticity. Through RNA sequencing, metabolomic, integrated bioinformatics, qRT-PCR, cellular and physiological analyses, we identify that maternal undernutrition leads to dysfunction in MAPK cell signalling and glycolytic metabolic pathways. We propose that these cells provide a valuable resource for DOHaD mechanistic studies, and can be used to generate *in vitro* models for studying developmental programming events in early embryogenesis or in specific organs through directed differentiation or organoid cultures.

## Materials and Methods

### Animal Treatment, ESC Line Derivation

Animal procedures were conducted under UK Home Office project license and local ethics approval (University of Southampton). C57BL/6 females (7–9 week) were mated naturally overnight with C57BL/6 males and plug positive females were housed individually the following morning and assigned randomly to either control normal protein diet (18% casein, NPD) or isocaloric low protein diet (9% casein, LPD) supplied by Special Dietary Services Ltd, UK, and fed *ad libitum* until embryonic day 3.5 (E3.5); diet composition has been described elsewhere [7]. Embryos were collected at the blastocyst stage after cervical dislocation and uterine flushing with H6 medium with 4 mg/ml BSA (H6 + BSA) [19]. Blastocysts were placed onto a freshly-prepared feeder layer of mitotically inactivated mouse embryonic fibroblasts (MEFs) prepared using standard procedures [20] in ES medium (knockout-DMEM [Gibco, 10829] supplemented with 15% knockout serum replacement (KSR) [Gibco, 10828], non-essential amino acids [Gibco, 11140], 1 mM sodium pyruvate [Gibco, 11360], 100 µM 2-mercaptoethanol [Sigma, M7522], 2 mM glutamine, penicillin [50 U/ml]/ streptomycin [50 µg/ml] (Gibco, 10,378) and 1000 U/ml of leukaemia inhibitory factor (LIF)), incubated at 37 °C in 5% CO_2_. After substantial outgrowth of the inner cell mass (ICM; 2.5 days culture), ICM-derived clumps from individual embryos were dislodged using a drawn capillary tube and mechanically dissociated into single-cell and small-cell aggregates in 0.05% trypsin–EDTA. Disaggregated cells were seeded onto fresh feeder layers on 96 well plates and cultured in ES medium. Viable ESC colonies were primarily passaged at day 3.5, expanded by trypsin passaging at 3-day intervals to reach confluence on a 100 mm dish. ESC lines were frozen after ~ 24 days in culture, passage number (PN) 5–7.

Sex determination of ESC lines was carried out after feeder cell depletion, by amplification of Y chromosome *Sry* and *Zfy* and control X chromosome *DXNds3* [21, 22]. Male ESC lines were predominant (LPD 70.6%, NPD 80.0%). ESC lines were karyotyped after at least 10 passages by fixation and Giemsa staining with most showing euploid karyotype (40 chromosomes in > 50% metaphase spreads; (LPD 71.4%; NPD 80.0%). A total of 18 ESC lines were generated from LPD blastocysts from 11 mothers and 38 lines from NPD blastocysts from 11 mothers. Male normal karyotype cell lines, all from different mothers, were used in all analyses.

### RNA Sequencing and Data Analysis

mRNA sequencing of five LPD and five NPD ESC lines (PN 9–12) was performed by an Illumina platform. RNA was extracted from ESCs cultured in fibroblast-free media using an RNeasy mini kit (Qiagen, UK). RNA was quality checked using nanodrop, agarose gel electrophoresis and Agilent 2100 Bioanalyzer followed by enrichment using oligo(dT) beads. The resulting mRNA was fragmented randomly and used as the template for synthesis of first strand of cDNA using random hexamers primer. The second strand synthesis was carried out by removing the RNA template and synthesizing a replacement strand to generate double-stranded cDNA. A series of terminal repair was carried out by ligation and adapter-ligation. The double-stranded cDNA library was completed through size selection and PCR enrichment. The quality control and concentration of the library was assessed using Qubit 2.0, Agilent 2100 bioanalyzer and qRT-PCR. The qualified libraries were fed into Illumina sequencers after pooling according to their effective concentration and expected data volume. The original data obtained from the high throughput sequencing platforms were transformed into sequenced reads by base calling. Reads were then filtered for adaptor contamination using cutadapt [23] and filtered such that at least 90% of bases of each read had a quality score > 20. Raw RNA sequencing data has been submitted to GEO repository (Accession number GSE216108).

The program Salmon was used for quantifying abundances of transcripts from RNA-sequencing data, or more generally of target sequences using high-throughput sequencing reads [24]. The index for Mus_musculus.GRCm38.88_transcripts.fa was built in Salmon using the default parameters. The program Wasabi was used to prepare Salmon output for downstream processing by Sleuth. Bootstrapping of the quantification was performed iteratively for 100 rounds. Resulting counts were then normalized through the transcripts per kilobase million (TPM) method. Differential expression analysis was carried out using the software package Sleuth (v0.30.0) using TPM normalized counts for each cell type. Gene Set Enrichment Analysis (GSEA) was carried out on the Broad Institute website (http://www.broad.mit.edu/gsea) using Hallmark Gene Sets of Molecular Signatures Database (MSigDB) collection because it represents 50 well-defined, large-scale biological processes that are applicable to the widest range of potential cellular responses [25]. The mouse genes were mapped to human genes before carrying out the analysis. A ranking metric was assigned to all the annotated genes using their P-values and fold change [26]. GSEA calculates unique enrichment score for each gene set that reflects the degree to which a gene set is overrepresented at either end of this continuum.

### Metabolomics and Data Analysis

Seven ESC lines were cultured (in duplicate) from each dietary group (PN 10–12), and expanded to 70–80% confluency in serum-free ES medium (KnockOut™ DMEM, LIF, 15% KO/SR), counted using trypan blue, flash frozen in liquid nitrogen as 50–100 μl packed cell pellets and stored at -80 °C. Samples were further processed using an automated MicroLab STAR® system (Hamilton) and analysed commercially (Metabolon Inc., NC USA) using ultra-performance liquid chromatography (UPLC, Waters ACQUITY) and a high-resolution tandem mass spectrometry (MS/MS, Thermo Scientific Q-Exactive) platform. Curated relative ion intensity data were normalised by both protein concentration (Bradford assay) and cell counts (trypan blue assay) analysis, and missing values were imputed to ensure improved data quality and to preserve biological variability of the samples. Metabolite values were normalised (i.e. rescaled) according to the median and log-transformed and back-transformed (as simple geometric means) and significance was evaluated by the Welch’s 2-sample t-test. The dataset was also adjusted for false discovery rate (FDR, q-value).

### Integrated Metabolomics and Transcriptomics Analysis

Paintomics version 4 was used to carry out integrated transcriptomics and metabolomics analysis using our two datasets [27]. The program integrates metabolite and transcript abundances and significances and maps them onto organism-specific Kyoto Encyclopedia of Genes and Genomes (KEGG) maps. All the annotated transcripts and metabolites with their fold-change values were imported and a list of the significant transcripts (*P* < 0.05) and metabolites (*P* < 0.05) were used as feature for respective analysis. The program computed P-values for individual transcriptomics and metabolomics analysis as well as combined P-values. The joint pathway enrichment P-value considering both the omics data was computed by applying the Fisher combined probability test. This is done by assigning a weight to transcriptomics and metabolomics that is the percentage of features mapped to pathways in that omic.

### Quantitative Real-time PCR

RNA was extracted as for above RNA sequencing for quantitative real-time PCR (qRT-PCR). cDNA was prepared using the Verso cDNA synthesis Kit (Thermo Fisher Scientific, Waltham, USA). A mix comprising 2 μl 5 × cDNA synthesis buffer, 1 μl deoxyribonucleotides triphosphate (dNTPs), 0.5 μl random hexamers, 0.5 μl oligo (dT) primers and 0.5 μl Verso enzyme mix was added to 150 ng RNA. The mix was then incubated at 42 °C for 60 min, followed by enzyme inactivation at 95 °C for 2 min as for manual instructions. The resulting cDNA was diluted 1:5 in Tris–EDTA (TE) buffer (Thermo Fisher Scientific) and stored at -20 °C until use. Primers used for qRT-PCR were designed using the online tools found in the Primer3 website (http://biotools.umassmed.edu/bioapps/primer3_www.cgi) and were designed to produce amplification products < 300 bp, and to have a melting temperature of 60 ± 1 °C (Supplementary Table 1). Primers were manufactured by Eurofins Genomics (Edersberg, Germany). For each sample, 1.5 ng of cDNA was used for qRT-PCR using a CFX96 RT instrument (Bio-Rad, Hercules, USA). Analysis of samples was performed in duplicate with each 10 μl reaction composed by 3 μl iTaq Universal SYBR Green Supermix (Bio-Rad), 300 nM of each forward and reverse primer and 5 μl diluted cDNA (0.3 ng/μl). The qRT-PCR reaction involved polymerase activation and cDNA denaturation at 95 °C for 5 min, followed by 40 thermal cycles consisting of denaturation at 95 °C for 5 s and primers annealing and extension at 60 °C for 30 s. Fluorescence was measured at the end of every cycle. Upon completion of thermal cycling, melting curve analysis was performed to confirm reaction specificity. Bio-Rad CFX Maestro software (Bio-Rad) was used to perform baseline subtraction and determine the threshold cycle (Ct). Normalization of expression of gene of interest to the selected reference genes (*Sdha*, *Tbp* and *Tuba-1*; GeNorm) was performed applying the formula for the calculation of the ΔCt. Data normalization and ΔΔCt calculations were performed using the CFX Maestro software (Bio-Rad). The expression fold change difference was calculated as 2 ^^(− ∆∆Ct)^ For pluripotency gene expression analysis, cDNA was synthesised by random priming from 250 ng RNA using the Improm- IITM Reverse Transcriptase System (Promega, UK) kit, amplification was conducted using a Chromo4 Real-time Detector (BioRad, UK) with Opticon Monitor v3.1 software, and amplified target genes were quantified following the SYBR-green precision mastermix (PrimerDesign, UK) protocol and using the same reference genes as above.

### ESC Proliferation and Cell Cycling

Proliferation was determined at early (PN 4–5) and later (PN 9–11) passages over 96 h in ES medium (2,500 cells in 150 µl/ well with MEFs), replaced daily, using conventional haemocytometer counts. Cell viability was determined using trypan blue (Sigma) exclusion in proliferation assays. Cell cycling was assessed after 24 h culture following 70% ethanol fixation of asynchronous ESCs, staining with propidium iodide (PI, 10 µg/ml PI with 250 µg/ml RNase, Sigma) and flow cytometric analysis (FACSCalibur, BD Biosciences) to determine proportion of G0/G1, S phase, and G2/M ESCs by mathematical deconvolution of DNA histograms using FlowJo 7.6.5 software (TreeStar, Inc., San Carols, CA). ESCs were also synchronised using nocodazole (80 ng/ml, Sigma) in ES medium for 6 h before release and subsequent PI staining and flow cytometry. S-phase cycling was also assessed after 24 h culture (1 × 10^5^ cells) in ES medium by BrdU incorporation (10 µM BrdU, 20 or 60 min), fixation in 70% ethanol, immunodetection of BrdU using mouse anti-BrdU antibody (1:100; Vector labs, VPB209) and secondary anti-mouse Alexa Fluor 488 (1:200, Molecular Probes), followed by PI staining and flow cytometry.

### ESC Survival and Apoptosis

ESCs (4 × 10^4^ cells) on MEFs in ES medium were harvested and trypsinized after 24, 48 and 72 h culture, stained with Annexin V-FITC and PI (100 µg/ml) in Annexin V binding buffer (10 mM HEPES pH 7.4, 140 mM NaCl, 2.5 mM CaCl_2_) for 30 min at 4 °C in the dark. For flow cytometric analysis, 20,000 cell events were recorded using a FACSCalibur flow cytometer (BD Biosciences). Data was assessed using Cell Quest Pro software (BD Biosciences). Forward scatter (indicating cell size) and side scatter (indicating cell granularity) gates were set so as to exclude MEF cells and debris. The percentage of apoptotic cells was determined by generating a dual-colour dot plot (Annexin V-FITC (FL-1) vs. PI (FL-2)) and then setting a quadrant marker based on unstained and single-labelled control samples.

### ESC Metabolic Activity

Measurement of glycolytic enzyme activity included two key allosteric enzymes, phosphofructokinase (PFK) and hexokinase (HK), assessed in lysates of fourteen (7 NPD and 7 LPD) ESC lines using colorimetric assay kits (Abcam, ab155898 and ab136957, respectively; same lot numbers throughout) according to manufacturer’s instructions. Two million live cells were diluted in 200 μl assay buffer, homogenized in UV-irradiated tubes on ice, centrifuged (12,000 rpm, 5 min, 4˚C), supernatants filter centrifuged (0.2 µm VectaSpin Micro centrifuge tube filter Z338591, Sigma Aldrich) and aliquots (20–30 μl) stored at -20 °C before analysis (duplicate samples, 0.5 µl, ~ 5 K cells) using Infinite F200 PRO (Tecan) microplate reader. Enzyme activity was normalised to cell counts and protein concentration (Bicinchoninic Acid (BCA) Assay) using the microplate reader.

### Immunofluorescence

Blastocysts and ESC lines were fixed in 4% paraformaldehyde, washed in PBS-Tween-20 (PBS-T, 0.05%), blocked and permeabilised (PBS with 0.3% Triton X-100 and 5% donkey serum) for 45 min, washed in PBS, incubated overnight at 4 °C with primary antibody (Supplementary Table 2) in PBS with 1% BSA and 0.3% Triton X-100, washed in PBS-T and incubated in secondary antibody conjugated with Alexa Fluor 488-, 546-, or 647- (Molecular Probes; 1:200 dilution) in PBS with 1% BSA and 0.3% Triton X-100 for 1 h at room temperature, and washed with PBS-T containing DAPI (0.2 µg/ml) in last wash. Samples were viewed on a Leica TCS SP5 confocal microscope and images analysed using Volocity software (PerkinElmer).

### Western Blotting

ESCs were grown feeder-free (3 × 10^5^ cells) and cell lysates prepared at 24, 48 and 72 h by incubation for 20 min with Onyx lysis buffer (20 mM Tris–HCl, pH 7.4, 135 mM NaCl, 1.5 mM MgCl_2_, 1 mM ethyleneglycoltetraacetic acid (EGTA), 1% TX-100, 10% glycerol) supplemented with both a protease inhibitor cocktail (Sigma. 1:100) and phosphatase inhibitors (2 mM Na_3_VO_4_, and 50 mM NaF). Reduced samples were fractionated by SDS-PAGE on pre-cast 4–12% NuPAGE® Bis–Tris polyacrylamide gels (Invitrogen). After separation, proteins were transferred to a methanol-activated Polyvinylidene Fluoride membrane (Immobilon-FL; Millipore) by overnight electro-elution. Membranes were blocked with 5% (w/v) non-fat dry milk in Tris-Buffered Saline (TBS) + 0.05% Tween20 (TBS-T) for 1 h at room temperature with constant agitation. Membranes were washed with TBS-T and probed with primary antibodies (Supplementary Table 2) overnight at 4 °C, washing and incubation with secondary antibody conjugated to IRDye™ 800CW (Rockland Inc. Molecular Probes) near-infrared (IR) fluorochrome for 1 h at room temperature in the dark. Following further washing, blots were scanned using the LI-COR Odyssey IR imaging system (LI-COR Biosciences). Integrated density values (IDVs) were obtained using the Odyssey software based on pixel intensity, and normalised to α-tubulin loading controls. Where membrane stripping was required, blots were incubated in stripping buffer (200 mM glycine, 3.5 mM SDS, 1% (v/v) Tween20, pH 2.2) for 10 min at room temperature, and then subsequently washed in PBS for 20 min and TBS-T for 10 min.

### Statistical Analyses

Statistical analysis of RNA sequencing, metabolomics and integrated Paintomics comprised tools available within the software programmes used and are referred to in the respective sections for generation of P values. The qRT-PCR, enzyme assays, and cellular studies were performed a minimum of three times and Minitab 16.1.0 or GraphPad Prism version 8.0 were used for statistical testing. Normality was tested using the Anderson–Darling or Shapiro–Wilk tests and considered normal when P > 0.05. Variance homogeneity was analysed using the F-test. Differences between dietary treatment groups were performed using the independent Student’s t-test or one way ANOVA (for normally distributed data), or Mann–Whitney rank sum test (for non-normal data sets). For the metabolic enzyme assay, differences were calculated using Welch’s t-test. All data are expressed as means ± SEM. *P* < 0.05 was considered statistically significant.

## Results

### LPD Reduces ESC Derivation Efficiency and Alters Expression of Pluripotency Markers in Blastocysts Undergoing ESC Transition

**Fig. 1:**
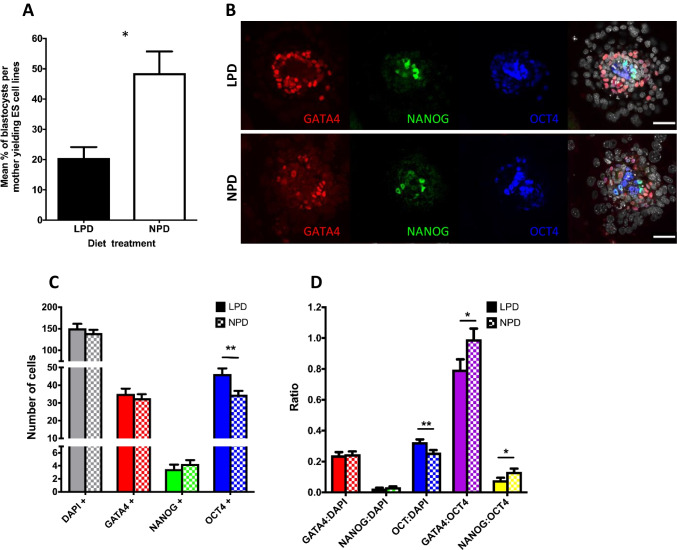
Effect of LPD on ESC line derivation and pluripotency. **A** Percentage (± SEM) of blastocysts yielding ESC lines. LPD, *n* = 100 blastocysts from 13 mothers; NPD, *n* = 93 blastocysts from 13 mothers. *P = 0.015. **B** Blastocyst outgrowth immunolabelling of GATA4, NANOG and OCT4 after culture for 2.5 d in ES cell medium. Scale bar = 50 µm. **C**, **D** Total number of ES cells (DAPI +) and proportion expressing specific lineage markers (**C**) and selected ratios (**D**) after 2.5 d culture in ES cell medium. LPD *n* = 29 blastocysts; NPD *n* = 35 blastocysts; data ± SEM; **P < 0.01, *P < 0.05

Similar numbers of blastocysts were isolated from LPD-fed and NPD control mothers (LPD 6.3 ± 0.44; NPD 6.5 ± 0.29; 16 mothers for each) which subsequently hatched from the zona pellucida and developed at similar rates during culture for ESC line derivation in the presence of leukemia inhibitory factor (LIF) and knockout serum replacement (KSR). However, fewer ESC lines were generated from LPD blastocysts (20%) compared to NPD blastocysts (49%; *P* = 0.016) (Fig. 1A). To see whether this distinction reflected changes in inner cell mass (ICM) pluripotency, we analysed the numbers of cells expressing the pluripotency markers NANOG and OCT4 and the primitive endoderm (PE) marker, GATA4, in blastocysts both immediately after collection at E3.5 (Supplementary Fig. 1A) and in outgrowths formed during subsequent 2.5 days culture in ES medium (Fig. 1B). No difference in the proportion of cells stained for NANOG, OCT4 or GATA4 was found in E3.5 blastocysts between treatments (Supplementary Fig. 1B), however, LPD outgrowths showed an increase in OCT4 positive cells (LPD 46.31 ± 3.21 vs. NPD 34.66 ± 2.16; *P* = 0.0029) while NANOG (~ 3–4 cells) and GATA4 (~ 30–40 cells) staining cells were comparable between groups (Fig. 1C). This resulted in LPD embryo outgrowths having a reduced NANOG:OCT4 ratio (LPD 0.079 ± 0.015 vs. NPD 0.134 ± 0.027; *P* = 0.044) and GATA4:OCT4 ratio (LPD 0.796 ± 0.067 vs. NPD 0.992 ± 0.070; *P* = 0.049) (Fig. 1D) and decreased the percentage of LPD outgrowths containing GATA4 stained cells (LPD 61.76% vs. NPD 88.24%). However, established LPD and NPD ESC lines exhibited equivalent expression of the three pluripotency factors *Nanog*, *Oct4* and *Sox2* at either mRNA or protein levels (Supplementary Fig. 1C, D).

To investigate biological distinctions between established LPD and NPD cell lines at low passage number, we used a multi-omics approach comprising RNA sequencing (summarised Supplementary Fig. 2; Table 1), metabolomics (summarised Supplementary Fig. 3; Table 2) and integrated analysis using PaintOmics (summarised Supplementary Fig. 4; Table 3), together with supportive molecular and cellular studies. Two main distinctions were revealed as presented below.

**Table 1:** Significant transcripts with FDR cut-off of < 0.1 following RNA sequencing

Transcript ID	Emsembl_gene	Gene	p-values	q-values	b (fold change)	se_b
ENSMUST00000096350.10	ENSMUSG00000042622	*Maff*	3.36E-11	1.47E-06	1.60228302	0.24167406
ENSMUST00000010211.6	ENSMUSG00000010067	*Rassf1*	1.95E-10	4.27E-06	0.73153193	0.11493014
ENSMUST00000103231.4	ENSMUSG00000029019	*Nppb*	9.70E-10	1.41E-05	1.73344337	0.28350733
ENSMUST00000216035.1	ENSMUSG00000032085	*Tagln*	7.38E-09	8.07E-05	1.97552366	0.34167052
ENSMUST00000161576.7	ENSMUSG00000031770	*Herpud1*	7.58E-07	0.00662829	0.55590104	0.11239779
ENSMUST00000095267.5	ENSMUSG00000071076	*Jund*	4.20E-06	0.03058265	0.3402554	0.07394704
ENSMUST00000109605.4	ENSMUSG00000042406	*Atf4*	6.64E-06	0.03628993	0.36337672	0.08066325
ENSMUST00000079780.9	ENSMUSG00000022686	*B3gnt5*	6.57E-06	0.03628993	0.42050915	0.09330008
ENSMUST00000005279.7	ENSMUSG00000005148	*Klf5*	1.90E-05	0.05558587	0.37719115	0.08821177
ENSMUST00000133033.1	ENSMUSG00000029455	*Aldh2*	1.79E-05	0.05558587	−1.0280612	0.23968415
ENSMUST00000231053.1	ENSMUSG00000055065	*Ddx17*	1.91E-05	0.05558587	−0.5595783	0.13088151
ENSMUST00000171581.1	ENSMUSG00000068206	*Pick1*	1.56E-05	0.05558587	−0.8333884	0.19289044
ENSMUST00000049836.13	ENSMUSG00000042331	*Specc1*	1.74E-05	0.05558587	0.68492738	0.15943239
ENSMUST00000030202.13	ENSMUSG00000028480	*Glipr2*	1.81E-05	0.05558587	0.33158862	0.07735129
ENSMUST00000206603.1	ENSMUSG00000036427	*Gpi1*	1.42E-05	0.05558587	−0.8982733	0.20695589
ENSMUST00000107362.9	ENSMUSG00000030530	*Furin*	2.28E-05	0.06216078	4.5955826	1.0848852
ENSMUST00000120524.1	ENSMUSG00000024736	*Tmem132a*	3.14E-05	0.07867326	−0.8732329	0.20975085
ENSMUST00000039926.9	ENSMUSG00000037887	*Dusp8*	3.24E-05	0.07867326	1.41597804	0.3407126
ENSMUST00000164773.1	ENSMUSG00000020205	*Phlda1*	4.05E-05	0.08572356	1.09134467	0.26588093
ENSMUST00000167495.7	ENSMUSG00000022185	*Acin1*	4.31E-05	0.08572356	−0.5157574	0.12610319
ENSMUST00000165027.8	ENSMUSG00000001627	*Ifrd1*	4.26E-05	0.08572356	0.40414202	0.09874008
ENSMUST00000029842.8	ENSMUSG00000028191	*Bcl10*	3.75E-05	0.08572356	0.359327	0.08716396
ENSMUST00000216891.1	ENSMUSG00000009741	*Ubp1*	4.68E-05	0.08893541	−0.5684329	0.13962698
ENSMUST00000000080.7	ENSMUSG00000000078	*Klf6*	5.25E-05	0.09565782	0.60671325	0.15002368

**Table 2:** Metabolomics list of metabolites showing significant (*P* < 0.05) or trend (*P* < 0.1) LPD/NPD fold change

Super pathway	Sub pathway	Biochemical	LPD/NPD fold change	P value
Amino acid	Glycine, Serine and Threonine	threonine	0.86	0.0796
	Alanine and Aspartate	asparagine	1.29	0.0289
	Methionine, Cysteine, SAM and Taurine	N-formylmethionine	0.89	0.0590
	Urea cycle; Arginine and Proline	dimethylarginine (SDMA + ADMA)	1.37	0.0797
		trans-4-hydroxyproline	0.78	0.0391
	Polyamine	spermidine	0.73	0.0470
	Glutathione	S-methylglutathione	0.70	0.0194
Carbohydrate	Glycolysis, Gluconeogenesis, and Pyruvate	glucose 6-phosphate	2.17	0.0354
		fructose-6-phosphate	1.63	0.0152
		glycerate	1.25	0.0386
	Fructose, Mannose and Galactose	mannose-6-phosphate	2.03	0.0425
	Nucleotide Sugar	glucuronate 1-phosphate	0.81	0.0299
	Aminosugar	glucosamine-6-phosphate	1.86	0.0572
Lipid	Medium Chain Fatty Acid	caproate (6:0)	0.68	0.0912
	Long Chain Fatty Acid	myristoleate (14:1n5)	1.30	0.0114
		palmitoleate (16:1n7)	1.33	0.0191
		10-heptadecenoate (17:1n7)	1.23	0.0638
		10-nonadecenoate (19:1n9)	1.20	0.0825
	Polyunsaturated Fatty Acid (n3 and n6)	eicosapentaenoate (EPA; 20:5n3)	1.38	0.0957
		docosapentaenoate (n3 DPA; 22:5n3)	1.30	0.0581
		docosahexaenoate (DHA; 22:6n3)	1.40	0.0919
		linoleate (18:2n6)	1.29	0.0190
		linolenate [alpha or gamma; (18:3n3 or 6)]	1.37	0.0151
		dihomo-linolenate (20:3n3 or n6)	1.34	0.0425
		adrenate (22:4n6)	1.42	0.0682
	Fatty Acid (Acyl Carnitine)	stearoylcarnitine	0.75	0.0625
	Endocannabinoid	stearoyl ethanolamide	1.26	0.0828
		linoleoyl ethanolamide	1.33	0.0335
	Phospholipid	glycerophosphoethanolamine	1.46	0.0674
		1-palmitoleoyl-2-linoleoyl-GPC (16:1/18:2)	1.23	0.0855
		1-palmitoleoyl-2-linolenoyl-GPC (16:1/18:3)	1.33	0.0635
		1-oleoyl-2-linoleoyl-GPE (18:1/18:2)	1.20	0.0824
	Lysolipid	1-palmitoleoyl-GPC (16:1)	1.40	0.0238
		1-oleoyl-GPE (18:1)	1.34	0.0968
		1-linoleoyl-GPE (18:2)	1.32	0.0927
		1-oleoyl-GPI (18:1)	1.32	0.0422
		1-linoleoyl-GPI (18:2)	1.54	0.0436
		1-stearoyl-GPS (18:0)	1.39	0.0368
		1-oleoyl-GPS (18:1)	1.38	0.0780
		1-palmitoyl-GPS (16:0)	1.57	0.0209
	Lysoplasmalogen	1-(1-enyl-palmitoyl)-GPE (P-16:0)	1.34	0.0925
		1-(1-enyl-oleoyl)-GPE (P-18:1)	1.46	0.0568
	Sphingolipid	N-palmitoyl-sphinganine (d18:0/16:0)	1.80	0.0220
		sphinganine	1.71	0.0717
		palmitoyl dihydrosphingomyelin (d18:0/16:0)	1.52	0.0221
		glycosyl-N-palmitoyl-sphingosine	0.82	0.0157
	Sterol	campesterol	1.69	0.0082
Nucleotide	Purine with Adenine	adenosine 5'-diphosphate (ADP)	0.53	0.0798
	Pyrimidine with Uracil	uridine 5'-monophosphate (UMP)	0.73	0.0461
		2'-deoxyuridine	1.17	0.0941
	Pyrimidine withThymine	thymidine	0.71	0.0471
		thymine	1.43	0.0289
Cofactors and Vitamins	Ascorbate and Aldarate	ascorbate (Vitamin C)	1.47	0.0305
		threonate	1.40	0.0276
Xenobiotics	Chemical	trizma acetate	0.76	0.0791

Data based upon 7 duplicated, protein-normalised samples each of LPD and NPD ESC lines (passage number 10–12)

**Table 3:** Significant pathways in the integrated transcriptomics and metabolomics analysis using PaintOmics

Pathway name	Unique genes	Unique metabolites	Significance tests		
			Transcriptomics	Metabolomics	Combined p-values*
MAPK signaling pathway	248		0.007813943	–	0.007813943
Glycolysis / Gluconeogenesis	56	9	0.003744399	0.295303567	0.00863274
Ascorbate and aldarate metabolism	9	10	0.014901416	0.09949872	0.011140662
Pentose phosphate pathway	27	11	0.044068364	0.082073827	0.023951382
Protein processing in endoplasmic reticulum	158		0.027200695	–	0.027200695
Glycosphingolipid biosynthesis—lacto and neolacto series	22		0.03605053	–	0.03605053
Hippo signaling pathway—multiple species	25		0.04086893	–	0.04086893
Starch and sucrose metabolism	22	4	0.03605053	0.207021198	0.044016361

^*^ The combined p-values were computed by applying the Fisher combined probability test

### LPD Impairs MAPK Network Signalling in ESCs Associated with Enhanced Cellular Stress and Apoptosis

**Fig. 2:**
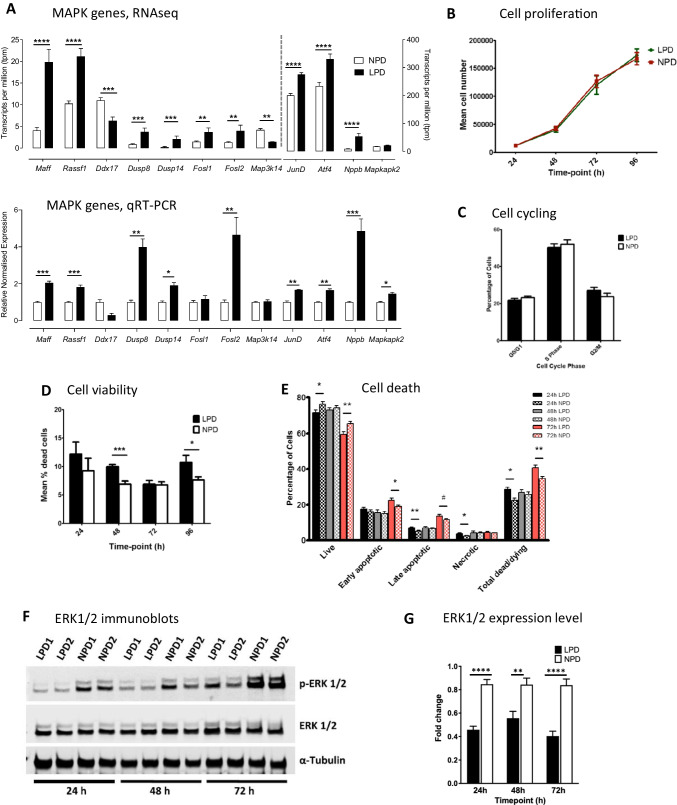
LPD causes impaired MAPK signalling with reduced survival and increased apoptosis in ESC lines.** A** RNAseq above and qRT-PCR analysis below of most differentially expressed MAPK components in undifferentiated LPD and NPD ESC lines (PN 8–12; *n* = 5) cultured in standard mESC medium (KnockOut DMEM + LIF + KO/SR). qPCR gene expression is normalised with 3 house keeping genes *Sdha*, *Tbp* and *Tuba-1*. Here, * = *P* < 0.05, ** = *P* < 0.01, *** = *P* < 0.001 and **** = *P* < 0.0001. **B** Proliferation rate of LPD and NPD ESC lines (P9-12) over 24 h intervals using trypan blue exclusion and haemocytometer counting. Data from 8 LPD or 9 NPD lines with mean ± SEM, each analysed in triplicate. **C** Cell cycle distribution of exponentially growing ESC lines (P10-12) 24 h after seeding, assayed by DNA quantitation using flow cytometry following PI staining and FlowJo software. Data from 10 ESC lines per treatment with mean ± SEM, each analysed in triplicate. **D** Cell viability assay. Mean percentage (± SEM) of dead cells from Trypan blue exclusion assay over 96 h culture (PN 11–13). LPD *n* = 8 ESC lines from 8 mothers; NPD *n* = 9 ESC lines from 7 mothers; ***P < 0.001, *P < 0.05. **E** Cell death assay. Percentage (± SEM) ESC lines (PN 11–12) after culture, annexin V and PI staining and flow cytometry discriminated as live, apoptotic and necrotic. LPD *n* = 8, 6, 7 lines at 24, 48 and 72 h; NPD *n* = 10, 9, 6 lines at 24, 48 and 72 h; **P < 0.01, *P < 0.05, #*P* < 0.1. **F**, **G** ERK1/2 immunoblots (**F**) and quantitation (p/total) (**G**) of ESC lines cultured up to 72 h. 13 + ESC lines per diet from minimum of 3 blots (independent experiments); α-tubulin used as loading control; ****P < 0.0001, **P = 0.0016. For antibody sources, see Supplementary Table 2

RNA sequencing indicated an overall similar phenotype between LPD and NPD ESC lines (Supplemetary Fig. 2A) but with differences in transcript expression identified in volcano plot (Supplementary Fig. 2B) and with 24 transcripts passing false discovery rate (FDR) at < 0.1 (listed Table 1) and 8 passing FDR at < 0.05 further visualised by heat map analysis (Supplementary Fig. 2C). Gene set enrichment analysis (GSEA) indicated enrichment of several pathways in LPD related to inflammation, energy metabolism and survival, collectively suggesting broad stress effects (Supplementary Fig. 2D). Three out of eight of the FDR < 0.05 transcripts, *Maff*, *Atf4*, *JunD*, were increased in LPD (all *P* < 0.0001) and are associated with the Mitogen-activated protein kinase (MAPK) pathway, a network of serial kinases with downstream effectors that target genes to coordinate cell proliferation, differentiation, apoptosis and stress response [28, 29]. The MAPK network was also the top significant class identified in PaintOmics, an integrated analysis of our transcriptomics and metabolomics datasets (Supplementary Fig. 4; Table 3).

RNA sequencing was validated by qRT-PCR for MAPK associated components. The most significant genes in RNA sequencing included upregulation of MAPK effectors *Maff* (*P* < 0.0001), *Rassf1* (*P* < 0.0001), *JunD* (*P* < 0.0001), *Atf4* (*P* < 0.0001), *Dusp8* (*P* < 0.01), *Dusp14* (*P* < 0.01) and *Fosl2* (*P* < 0.01) in LPD which were confirmed by qRT-PCR (Fig. 2A). *Ddx17* (*P* < 0.001)and *Map3k14* (*P* < 0.01) were significantly reduced in RNA sequencing but not significant in qRT-PCR. Similarly, *Fosl1* was upregulated in both RNA sequencing and qRT-PCR in LPD but was only significantly changed in RNA sequencing (*P* < 0.01). *Mapkap2* was significantly increased in LPD cells (*P* < 0.05) in qRT-PCR but the increase was deemed non-significant in RNA sequencing (Fig. 2A). We also included *Nppb* which encodes brain natriuretic peptide within the MAPK effectors since the hormone mediates the MAPK pathway to regulate cardiac response to stress [30]. Thus, LPD ESC lines exhibited upregulation of *Nppb* in RNA sequencing (*P* < 0.0001) and confirmed by qRT-PCR (Fig. 2A).

Since MAPK signalling affects cell proliferation, this was measured for the ESC lines firstly during initial cell expansion at early passage number (PN 4–6) before cells were frozen, and secondly after cell expansion and freezing (PN 9–12). Viable cell counts comparing the change in cell numbers over 24, 48, 72 and 96 h after passage were determined and showed that the rate of increase was near identical between diet treatments at both early and late passages (Fig. 2B; Supplementary Fig. 5A). Cell cycling was assessed after 24 h of culture by propidium iodide (PI) staining and flow cytometry and showed similar proportions of G0/G1, S and G2/M cells in LPD and NPD lines (Fig. 2C). The rate of BrdU incorporation at 24 h to identify S-phase cells was also similar between LPD and NPD lines (Supplementary Fig. 5B). Lastly, ESCs synchronised using nocodazole and subsequently released displayed similar cell cycle characteristics across diet treatments (Supplementary Fig. 5C).

MAPK signalling is also critical in regulating cell survival and apoptosis [28, 29] and so this was evaluated next. Trypan blue staining of cells at 24–96 h after plating showed greater proportions of dead (trypan +) cells in LPD ESC clones (Fig. 2D) suggesting reduced survival. This was most evident at 48 h (LPD 10.01 ± 0.331%, NPD 6.93 ± 0.552%; *P* = 0.003) and 96 h (LPD 10.71 ± 1.256%, NPD 7.64 ± 0.572%; *P* < 0.05). This was substantiated by flow cytometric assessment of cell lines following FITC-Annexin V staining which binds to phosphatidylserine, externalised during early apoptosis, and PI for loss of cell integrity (late apoptosis). A representative density plot of AnnexinV-PI staining by flow cytometry is shown in Supplementary Fig. 5D. LPD ESC lines exhibited fewer live cells and increased early and late apoptosis and total dead cells over 72 h culture compared with NPD lines (*P* < 0.05 – 0.01) (Fig. 2E). Quantitative immunoblotting of ESC lines over 72 h culture was used to assess cell signalling activity that may explain the dietary-induced change in cell apoptosis. ESCs maintain their pluripotent state through LIF signalling which activates the STAT3 cascade to retain undifferentiated self-renewal and regulate cell survival, through activation of PKB/AKT signalling [31, 32]. We analysed activation of AKT (phosphorylation at ser493) and STAT3 (phosphorylation at tyr705) but neither indicated a change in activity or total levels between diet groups (Supplementary Fig. 6A, B). MAPK signalling is known to be induced in ESCs through ERK1/2 and the stress-related p38 pathways [31, 33–37]. We tested the activation of ERK1/2 (phosphorylation at thr202/tyr204) and p38 (phosphorylation at thr180/tyr182) and found that whilst both had similar total protein levels across diet groups, ERK1/2 activation, but not that of p38 (Supplementary Fig. 6C, D), was reduced by ~ 50% in LPD ESCs (*P* < 0.0016- < 0.00001 over 24–72 h culture) (Fig. 2F, G). Lastly, analyses of several components in the apoptotic pathway (PUMA, BimEL, Caspase 3, Cleaved caspase 3; [38]) showed no difference across diet treatment (Supplementary Fig. 6C, D). These data show that altered MAPK pathway activity through reduced ERK1/2 signalling in LPD ESC lines coincides with their increase in apoptosis and cell death rate and reduced derivation efficiency.

### LPD Impairs Glycolysis and Carbohydrate Metabolism in ESC Lines

Metabolomics was carried out on low passage (P10-12) LPD and NPD ESC lines and, whilst having a similar overlapping profile (Supplementary Fig. 3A, B), random forest analysis (Supplementary Fig. 3C) revealed changes in glycolytic and carbohydrate metabolism alongside changes in amino acid and fatty acid metabolism in LPD ESC lines (see Table 2 for list of changed metabolites). Glycolysis was the second-most significant pathway in the integrated PaintOmics analysis, alongside associated pentose phosphate and sugar metabolism pathways (Table 3; Supplementary Fig. 4).

**Fig. 3:**
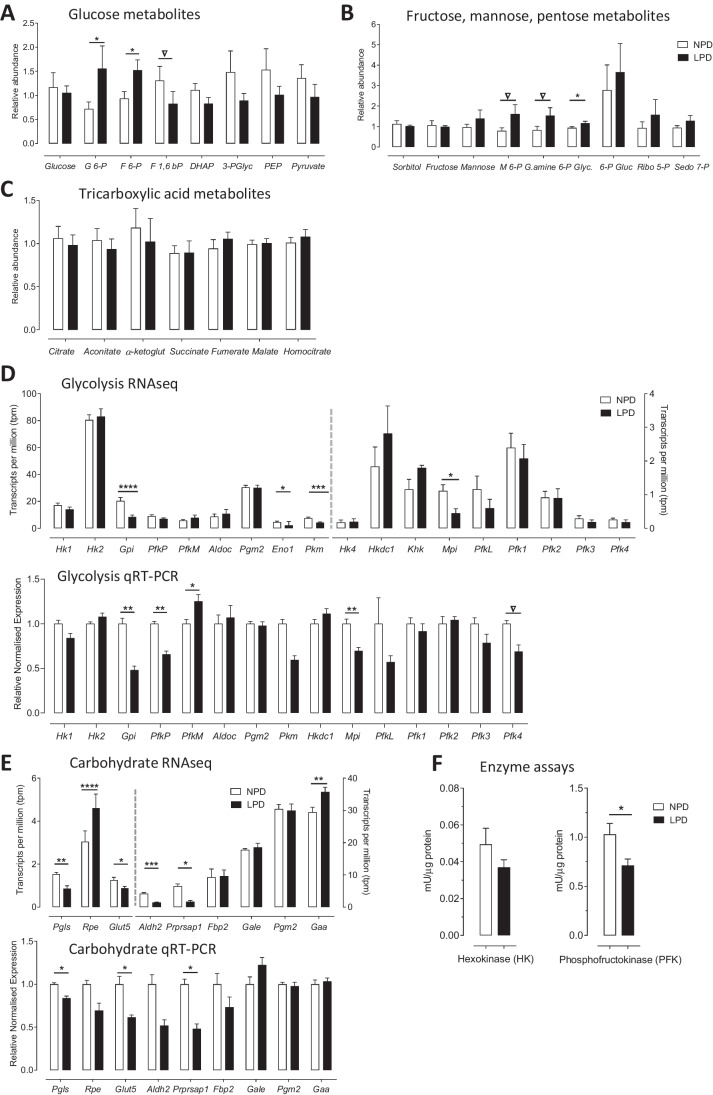
LPD impairs glycolytic and carbohydrate metabolism in undifferentiated LPD ESC lines.** A-C** Metabolomics analysis of mean concentrations (protein normalised) of key metabolites involved in **(A)** glycolysis, **(B)** fructose, mannose, pentose pathways, and **(C)** TCA pathways from lysates of undifferentiated male NPD and LPD mESCs (PN 10–12; *n* = 7) cultured in standard mESC medium (KnockOut DMEM + LIF + KO/SR). * *P* < 0.05, ▽ *P* < 0.1. G 6-P = glucose 6-phosphate; F 6-P = fructose 6-phosphate; F 1,6 bP = fructose 1,6-bisphosphate; DHAP = dihydroxyacetone phosphate; 3-PGlyc = 3-phosphoglycerate; PEP = phosphoenolpyruvate; M 6-P = mannose 6-phosphate; G.amine 6-P = glucosamine 6-phosphate; Glyc. = glycerate; 6-P Gluc = 6-phosphogluconate; Ribo 5-P = ribose 5-phosphate; Sedo 7-P = sedoheptulose-7-phosphate; -ketoglut = alpha-ketoglutarate. **D**, **E** RNAseq above and qRT-PCR analysis below of gene expression of glycolytic enzymes (**D**) and other carbohydrate metabolism regulators (**E**) in undifferentiated LPD and NPD ESC lines (PN 9–12; *n* = 5) cultured in standard mESC medium (KnockOut DMEM + LIF + KO/SR). qPCR gene expression is normalised with 3 house keeping genes *Sdha*, *Tbp* and *Tuba-1*. * = *P* < 0.05, ** = *P* < 0.01, *** = *P* < 0.001, **** = *P* < 0.0001, ▽ *P* < 0.1. *Hk1*, *Hk2*, *Hk4* = hexokinase 1,2 or 4; *Gpi* = glucose phosphate isomerase; *PfkP*, *PfkM, PfkL* = phosphofructokinase P, M or L variants; *Aldoc* = aldolase C; *Pkm* = pyruvate kinase M; *Hkdc1* = hexokinase domain containing 1; *Khk* = ketohexokinase; *Mpi* = mannose phosphate isomerase; *Pfk1-4* = phosphofructokinase 1–4 variants; *Pgls* = phosphogluconolactonase; *Rpe* = ribulose-phosphate 3-epimerase; *Glut5* (*Slc2a5)* = glucose transporter 5; *Aldh2* = aldehyde dehydrogenase 2; *Prprsap1* = phosphoribosyl pyrophosphate synthetase-associated protein 1; *Fbp2* = fructose-bisphosphatase 2; *Gale* = UDP-galactose-4-epimerase; *Pgm2* = phosphoglucomutase 2; *Gaa* = acid alpha-glucosidase. **F** Glycolytic enzyme activity assays on hexokinase (HK) and phosphofructokinase (PFK) from LPD and NPD ESC lines. Data, normalised by protein (BCA Protein Assay), presented as means ± SEMs based on *n* = 6 for PFK and *n* = 7 for HK analysis. *P = 0.029

For glycolysis and carbohydrate metabolism, the metabolomics analysis of LPD ESC lines exhibited an increase in some upstream glucose-derived intermediates below glucose itself at either trend or significant levels including glucose 6-phosphate (G6-P; *P* = 0.044), fructose 6-phosphate (F6-P; *P* = 0.043) and mannose-6-phophate (M6-P; *P* = 0.090) (Fig. 3A, B). Moreover, the derivative of F6-P, glucosamine 6-phosphate (G.amine 6-P; *P* = 0.057) was also increased at trend level (Fig. 3B). Conversely, the downstream metabolites fructose 1,6-bisphosphate (F1,6 BP; *P* = 0.115), dihydroxyacetone phosphate (DHAP; *P* = 0.129), 3-phosphoglycerate (3-PGlyc; *P* = 0.788), phosphoenolpyruvate (PEP; *P* = 0.783) and pyruvate (*P* = 0.232) were at reduced mean levels but not individually significant (Fig. 3A). In contrast to glycolysis, minimal or no differences were identified in remaining pentose and tricarboxylic (TCA) pathway metabolites (Fig. 3B, C).

To explore mechanisms causing these metabolomic changes in LPD, expression of enzymes and other regulatory genes involved in the glycolysis pathway and sugar metabolism were analysed by RNA sequencing and qRT-PCR (Fig. 3D, E). From the top of the glycolytic pathway, expression of various hexokinase (*Hk1-4, Hkdc1, Khk*) isoforms, responsible for glucose conversion to G6-P, were unchanged both in RNA sequencing and qRT-PCR (Fig. 3D). However, glucose phosphate isomerase (*Gpi*; *P* < 0.0001) and mannose phosphate isomerase (*Mpi*; *P* < 0.05), responsible for catalysing G6-P and M6-P respectively to F6-P, were significantly reduced in LPD in RNA sequencing and confirmed by qRT-PCR (Fig. 3D). Next in the pathway, phosphofructokinase (*Pfk*) isoforms, responsible for F6-P breakdown, were unchanged in RNA sequencing but in qRT-PCR three of seven *Pfk* isoforms tested showed divergent changes, either increased (*PfkM*; *P* < 0.05) or decreased (*PfkP*; *P* < 0.01; *PfK4*; *P* < 0.1) expression in LPD (Fig. 3D). Other descending glycolytic enzymes in the pathway were unchanged including aldolase C (*Aldoc*), glyceraldehyde 3-phosphate dehydrogenase (*Gapdh*), phosphoglycerate kinase (*Pgk*) and phosphoglycerate mutase (*Pgm2*) (data not shown except *Aldoc* and *Pgm2*, Fig. 3D). However, the terminal enolase (*Eno1*; *P* < 0.05) catalysing PEP production, and pyruvate kinase (*Pkm*; *P* < 0.001) catalysing pyruvate production, were both reduced in LPD (Fig. 3D).

In addition to these changes in the direct glycolysis pathway, LPD also altered expression of select genes involved more broadly in carbohydrate metabolism. Thus, expression of pentose phosphate pathways genes, phosphogluconolactonase (*Pgls*; *P* < 0.01) and phosphoribosyl pyrophosphate synthetase-associated protein 1 (*Prpsap1*; *P* < 0.05) were reduced and ribulose-phosphate 3-epimerase (*Rpe*; *P* < 0.0001) increased in LPD while the glucose transporter *Glut5* (*Slc2a5* *P* < 0.05) was reduced (Fig. 3E). Aldehyde dehydrogenase 2 (*Aldh2*), involved in alcohol metabolism, was reduced (*P* < 0.001) and acid alpha-glucosidase (*Gaa*), involved in glycogen processing, increased (*P* < 0.01) in LPD in RNA sequencing but not confirmed by qRT-PCR (Fig. 3E).

The changes identified in glycolytic metabolites and associated gene expression following LPD, with accumulation of upstream metabolites coupled with reduced enzyme expression but with depleted metabolites downstream, is suggestive of a bottleneck in processing at the PFK step. Thus, enzyme activity assays were performed on both hexokinase (HK) glucose to G6-P conversion, and on phosphofructokinase (PFK) F6-P to F-1,6-bP conversion. Although no difference was observed in HK activity (*P* = 0.2), PFK activity in LPD lines was reduced (*P* = 0.03, Fig. 3F).

## Discussion

**Fig. 4:**
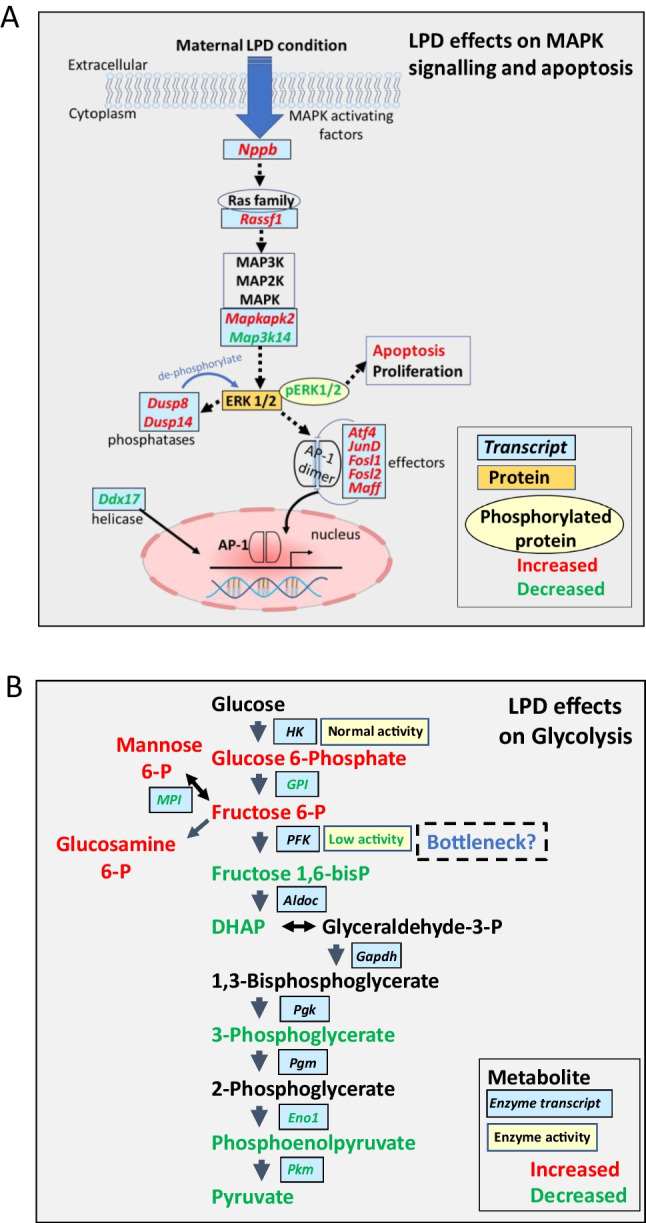
Summary diagrams of changes in MAPK pathway and glycolytic pathways in LPD vs NPD ESC lines. **A**. **MAPK.** Changes in relevant MAPK pathway components at transcript, protein and phosphorylated state indicating possible origin of increased apoptosis. Red indicates increased and green reduced levels in LPD based upon outcome of transcriptomic and cellular studies. See Fig. 2 and text for abbreviations. **B**. **Glycolysis.** Changes in relevant RNA expression, metabolite levels, and downstream cellular / physiological phenotype are shown. Red indicates increased and green reduced levels in LPD based upon transcriptomic, metabolomic and cellular studies indicative of bottleneck at PFK and shown by reduced activity. Some metabolites at mean rather than significant levels, see text. See Fig. 3 and text for abbreviations

Our characterisation of ESC lines derived from mothers fed LPD during the preimplantation period was conducted to identify early mechanisms of adverse developmental programming that can lead to increased risk of non-communicable diseases in later life [2, 4]. Analyses of LPD ESC lines compared with control NPD lines using RNA sequencing, metabolomics, integrated bioinformatics, qRT-PCR and cellular studies revealed two major dysfunctional phenotypic states comprising altered gene expression and signalling activity of members of the MAPK signalling network (summarised Fig. 4A) and changes in the expression and activity of the glycolytic energy metabolism pathway (summarised Fig. 4B). We also found reduced efficiency in the derivation of LPD ESC lines alongside more subtle and transient changes in expression of pluripotency markers in undifferentiated cells which may be linked to the MAPK phenotype. Also, omics approaches identified additional changes in signalling pathways (see, for example, Supplementary Fig. 2D and 4) that may further modulate phenotype which, while not corroborated here with additional studies, are discussed below to contextualise our outcomes.

MAPK signalling is a highly conserved network of serial kinases belonging to JNK, p38 and ERK classes responsive to environmental stimuli which phosphorylate downstream effectors to activate or repress expression of target genes that regulate fundamental cellular activities such as proliferation, differentiation, apoptosis and stress response [28, 29]. Whilst our RNA sequencing and qRT-PCR analyses showed only minimal changes in expression of specific MAPK kinases and accessory proteins (eg, *Map3k14*; *Mapkapk2*), pronounced and consistent changes were identified in downstream effectors regulating transcriptional activity. Thus, LPD lines displayed a significant upregulation of several MAPK transcripts including *JunD*, *Atf4*, *Fosl1*, *Fosl2* and *Maff* which collectively encode subcomponents of the activating protein-1 (AP-1) homo- or hetero-dimer transcription factor complex comprising the integrated stress response [39, 40]. The AP-1 dimerised elements bind to cAMP response element (CRE) sites to effect downstream expression associated with cell survival or apoptosis in response to various stresses including amino acid starvation, ER stress and oxidative stress [41–43]. We note that amino acid deficiency in the uterine fluid has been identified as an initiator of LPD programming in the mouse preimplantation embryo leading to later life health risk [11, 44] and so may be responsible causally for AP-1 transcript changes in LPD ESC lines. The combination and abundance of AP-1 dimerised partners can influence whether positive or negative regulation of downstream targets occurs leading to apoptotic or proliferative cell survival outcomes. For example, FOSL1 is expressed in tumour tissue where it promotes growth and differentiation rather than apoptosis [43].

In addition to increased expression of AP-1 mRNAs, LPD lines also expressed increased *Dusp8* and *Dusp14* mRNAs which encode members of the dual specificity phosphatase (DUSP) protein family that dephosphorylate MAPK kinases including ERKs as part of the regulation of stress response towards survival or apoptosis [45, 46]. LPD lines were also characterised by increased *Rassf1* expression which belongs to the RASSF protein family that links RAS to the MAPK pathway. As in above examples, RASSF has been shown to contribute to diverse cellular functions across the range from apoptotic to proliferative activities [47]. Further, LPD lines were downregulated in *Ddx17* which encodes multitasking RNA helicases that similarly modulate cellular activity from apoptosis to proliferation [48]. Lastly, LPD lines exhibited upregulation of *Nppb* encoding B-type natriuretic peptide (BNP) which contributes to MAPK-mediated stress response in cardiomyocytes [30]. BNP plays a role in cardiac development and elevated plasma levels associate with heart failure and myocardial infarction [49]. Maternal undernutrition can also cause elevated BNP and increased cardiac dysfunction during development [50] but effects mediated through proliferation or apoptosis are unknown.

To evaluate the functional implications of MAPK pathway modulation in LPD lines, we first examined cell proliferation and cell cycle control. In detailed studies, we were not able to identify any changes in the rate of proliferation over 96 h using early or later passage cells, nor were differences found in cell cycling stages using flow cytometry, BrdU incorporation, and synchronised cell populations. In contrast, analysis of cell survival and apoptosis showed LPD ESCs had increased proportion of dead cells within cultures over time, and, following flow cytometry, had fewer live cells and increased early and late apoptotic and dead cells. Thus, MAPK signalling changes in LPD lines more likely relate to poorer survival than regulation of cell proliferation. Moreover, the poorer rate of establishment of LPD lines is consistent with this interpretation.

To assess directly MAPK signalling, we quantified by immunoblotting the posttranslational state of MAPK classes, p38 and ERK, known to promote apoptosis in mESCs [35]. Whilst no change was found in p38 signalling, ERK1/2 was significantly dephosphorylated in LPD lines, representing relative ERK1/2 inactivation. This altered state is consistent with the increase in apoptosis and cell death found in LPD lines by flow cytometry but is likely to be measured since the posttranslational state of selected apoptotic markers in LPD lines was not altered. This distinction between the flow cytometry and apoptosis immunoblotting data may reflect the relatively small changes in apoptotic cell percentages identified (Fig. 2E), the relative extent of apoptotic protein expression and post-translational processing, or the timing of effector activation of proteins (eg, caspase-3) not synchronising with later morphological status of cells screened by flow cytometry (eg, Annexin V binding; PI internalisation). The altered MAPK and ERK1/2 phenotype is also consistent with our previous findings that LPD neural stem cells during fetal brain development are reduced in number partly through increased apoptosis [9].

The reduced ERK1/2 activity in LPD lines, in addition to the effects on cell survival and apoptosis, may also influence the observed minor change in pluripotency expression. Evidence indicates ERK signalling has a dual role in mESCs, with ERK activation promoting differentiation through destabilising nuclear pluripotency factors and its inhibition maintaining self-renewal [34, 51, 52], however, a minimal ERK activity is required to protect genome stability and pluripotency [53, 54]. Thus, we found LPD lines transiently possessed more cells expressing nuclear OCT4 protein during their derivation which is consistent with the reduced ERK1/2 activity.

The second major dysfunctional phenotype found in LPD ESC lines was associated with glycolytic energy metabolism, identified mainly by the metabolomics screen but supported by RNA sequencing, qRT-PCR and physiological datasets. Here, the upstream steps in the 10 enzyme pathway of cytoplasmic glycolytic metabolism from glucose to pyruvate were affected (summarised Fig. 4B). Whilst glucose levels were unchanged, the glycolytic intermediates G6-P and F6-P were both significantly increased in LPD ESC lines; similarly, the intermediates from mannose metabolism, M6-P which interconverts with F6-P, and its end-product G.amine 6-P, were also increased at trend level in LPD cells. RNA sequencing and qRT-PCR both showed that the expression of the key enzymes leading to F6-P production, namely *Gpi1* from G6-P and *Mpi* from M6-P, were both significantly reduced in LPD lines. In contrast to the elevated levels of upstream sugars, glycolytic metabolites downstream from F6-P showed some evidence of reduction in LPD. Thus, F1,6 BP, DHAP, 3-PGlyc, PEP and pyruvate were all reduced in LPD but not individually reaching significance. This glycolytic pattern (Fig. 4B) suggests a ‘bottleneck’ may occur in the pathway at F6-P, limiting downstream processing. F6-P metabolism to F1,6 BP is a rate-limiting step for glycolysis mediated through phosphofructokinase (PFK) whose activity is primarily regulated through dimer and tetramer formation and stabilisation of the protein [55]. Moreover, PFK expression and activity has been shown to be developmentally regulated from the embryo onwards [56, 57]. Genes encoding PFK and downstream enzymes in glucose metabolism were mainly unaltered in RNA sequencing or qRT-PCR although minimal *Pfk* isoforms were divergently changed and pyruvate kinase (*Pkm*; converting PEP to pyruvate) were downregulated in LPD, possibly contributing to impaired glycolysis. Thus, the putative F6-P bottleneck may reflect either transcriptional (*Pfkp*), translational, and/or posttranslational modification in PFK enzyme activity. PFK activity was indeed shown to be significantly reduced in LPD cell lines whilst HK activity which acts on glucose conversion to G6-P was unchanged.

These data therefore indicate LPD ESC lines show deficiency in glycolytic metabolism through reduced PFK activity possibly combined with reduced expression of *Gpi1*, *Mpi* and *Pkm*. There is also suggestion that the accumulation of upstream intermediates coupled with reduced PFK results in increased redirection of F6-P to G.amine 6-P and 6-P Gluc in the pentose pathway. How might these changes in glycolytic metabolism be mediated? Collectively, the impairment in PFK activity and glycolysis may reflect the altered maternal metabolism induced by LPD affecting the early embryo and ESC phenotype. LPD causes maternal hyperglycaemia through reduced circulating insulin and raised glucose at the time of blastocyst formation [11, 58]. Reduction in insulin signalling in different diabetic models has been shown to disturb glycolysis through reduction in PFK protein but not transcription [59, 60]. Moreover, metabolomic screening of adult cardiomyocytes under hypoinsulinaemic conditions revealed increased glycolytic intermediates upstream of PFK activity (F6-P, G6-P) and reduced downstream metabolites (fructose-1,6,bisphosphate [F 1,6 bP], 3-phosphoglycerate [3-PGlyc]) [59], closely mirroring the phenotype of LPD ESCs. Finally, maternal LPD has been reported to induce metabolic and mitochondrial dysfunction in early mouse embryos [61], a phenotype consistent with our data here. An increase in LPD fatty acids (listed Table 2) may be a further consequence of glycolytic impairment mediated by low insulin whereby maintaining energy supply requires a switch towards fatty acid metabolism as found in type 2 diabetic conditions [62].

Whilst we have focused on the phenotype changes in MAPK signalling and glycolytic metabolism, the RNA sequencing and Paintomics integrated analyses also found changes in other related signalling pathways, notably mammalian target of rapamycin complex 1 (mTORC1) signalling identified as an enriched gene set following GSEA (Supplementary Fig. 2D) and HIPPO signalling following string interaction Paintomics (Supplementary Fig. 4). Substantial crosstalk occurs across several signalling pathways in cells to fine-tune responses to specific environmental cues with, for example, integrated activity across MAPK, mTORC1 and HIPPO signalling following LIF stimulation [63]. Changes in mTORC1 activity commonly occur in DOHaD developmental models to coordinate growth with nutrient availability [64] including the current mouse LPD model [4]. An integrator role for HIPPO signalling has also been recognised coordinating input from metabolism and nutrient levels with cell contact patterns to regulate growth and differentiation [65]. Moreover, HIPPO signalling is directly sensitive to glycolytic activity and energy availability [65, 66]. Thus, the phenotype we describe for LPD ESC lines may reflect a combinatorial response across signalling pathways but further studies will be required to confirm.

### Conclusion

Our detailed characterisation of LPD ESC lines provides further support for their use in identifying early mechanisms of adverse developmental programming. We highlight two major changes in phenotype with (i) impairment of MAPK signalling associated with reduced cell survival and increased apoptosis and (ii) impairment in upstream glycolytic metabolism mediated in particular by reduced PFK activity. In the sequence of induction of adverse programming mediated through maternal protein restriction, these changes in ESC phenotype derive from the altered maternal metabolite environment in the vicinity of the embryo before implantation. However, the ubiquity of MAPK signalling and energy metabolism in regulating cellular function is such that future studies will be required to identify precisely how these conditions contribute to the step-wise programming of disease phenotype. For example, maternal LPD treatment is known to alter fetal and postnatal growth trajectory which in turn associates with adult disease risk [4] and influences the extent of cellular differentiation and apoptosis during development [9, 11] which may reflect altered MAPK signalling. Energy metabolism is also recognised as central to embryo survival and viability and a contributor to DOHaD mechanisms [67]. The combination of these signalling and metabolic effects may also be causally related. For example, in cancer cells, a non-glycolytic role for *Pfkp* has been identified to alter MAPK signalling activity [68]. These datasets therefore provide the basis for more directed *in vivo* and *in vitro* studies to understand mechanisms and preventative approaches to the developmental origin of disease.

## Supplementary Information

Below is the link to the electronic supplementary material.

Supplementary file1 (DOCX 21 KB) comprising Supplementary Tables 1, 2.

Supplementary file2 (PPTM 7104 KB) comprising Supplementary Figures 1-6.

## Data Availability

The RNA sequencing dataset has a GEO accession number GSE216108; the metabolomics dataset and other data presented are available from the corresponding author on reasonable request.
