# Supplementary material for: Maternal Undernutrition Induces Cell Signalling and Metabolic Dysfunction in Undifferentiated Mouse Embryonic Stem Cells
Source: Stem Cell Rev Rep. 2022 Dec 15;19(3):767–83. doi: 10.1007/s12015-022-10490-1 (PMC10070223; doi:10.1007/s12015-022-10490-1)
Supplement: Supplementary file 1 — Supplementary file1 (DOCX 21 KB) comprising Supplementary Tables 1, 2. [file 12015_2022_10490_MOESM1_ESM.docx]

**Supplementary Table 1**. Primers used for gene expression (qRT-PCR) studies. Here, Tv= transcript; RI= reverse intron; CM= computationally mapped; CA= canonical; NMD= nonsense mediated decay; PT= processed transcript.

|  | **Gene** | **Forward sequence** | **Reverse sequence** |
| --- | --- | --- | --- |
| **Pluripotency** | *Nanog* | TGCTTACAAGGGTCTGCTACTG | GAGGCAGGTCTTCAGAGGAA |
|  | *Oct4* | GTTGGAGAAGGTGGAACCAA | CTCCTTCTGCAGGGCTTTC |
|  | *Sox2* | TGGGCTCTGTGGTCAAGTC | TGATCATGTCCCGGAGGT |
|  |  |  |  |
| **Reference** | *Sdha* | TGTTCAGTTCCACCCCACA | TCTCCACGACACCCTTCTGT |
|  | *Tbp* | GGGAGAATCATGGACCAGAA | GATGGGAATTCCAGGAGTCA |
|  | *Tuba-1* | CTGGAACCCACGGTCATC | GTGGCCACGAGCATAGTTATT |
|  |  |  |  |
| **RNA seq. &** | *Akt3-202 (Tv)* | GGTTGGGTTCAGAAGAGGGG | AGCCTATGAATGAGCCATCTGT |
| **Metabolomics** | *Aldh2-203 (RI)* | CTTTATCCAGCCCACCGTGT | TCCTACCAAAAGCAGCAGGG |
|  | *Aldh2-205 (RI)* | TGGACCAGTGATGCAAATCCT | AGGATTTGCATCACTGG TCCA |
|  | *Aldoc-202 (CM)* | TTCTGGCTGCAGATGAGTCC | CGGCGATTCTCCTCAGTGTT |
|  | *Atf4-201(CA)* | AATGGCCGGCTATGGATGAT | ACATCCAATCTGTCCCGGAAA |
|  | *Ddx17-204 (CA)* | TCCTATCCTCATTGCCACGG | TTCTCTGTAGCCCCATTTGGT |
|  | *Dusp14-205 (CA)* | GCCGACAAGATCCACAGTGT | CAATACAGAGGGTGGCCGAG |
|  | *Dusp8-201(CA)* | GAGCCGTTTCATGCGTATCC | CAATGACTTGGCAGCTGGAC |
|  | *Fbp2-201 (CA)* | GAACTCACCCAGCTGCTCAA | TCCTGTCACATTCACGCTCC |
|  | *Fosl1-201(CA)* | CAGCAGCAGAAGTTCCACCT | GGTCCCAGGAAATGAGGCTG |
|  | *Fosl2-201(CA)* | CGGGAACTTTGACACCTCGT | GAGCCAGGCATATCTACCCG |
|  | *Gaa-203 (CA)* | CTGGAGGTGCTGATGGAGAC | TCTCCAGGGGCACTTCGTA |
|  | *Gale-202* | GGACATCTTGGACCAGGCAG | CCTTGAGGCCAGCAAAGTG |
|  | *Glipr2-201(CA)* | GAAGCCCAGCAGTATTCGGA | CGCAAGGTTCTCTCCACACT |
|  | *Glut5-201 (CA)* | CTGACGGTGTCCATGTTCCC | GCCCCTTTTCTGCCCAGTT |
|  | *Gnas-206* | CAGTAAGACCGAGGACCAGC | CAGGATCCTCATCTGCTTCACA |
|  | *Gpi1-203 (RI)* | ACCCACCTTCAATGCCC TTT | AGCTGCTCGAAGTGGTC AAA |
|  | *Gpi1-206 (NMD)* | TGCTGTGACCTCCCATG ATT | GACCAGTCAGTAGGGCC G |
|  | *Gpi1-211 (RI)* | CCTGTCTACGAACACGG CC | AGACTCGGCAAAGGACA CAC |
|  | *HK1-202/203 (CA)* | ACACCCCAGAGAACATCGTG | CTCCATGAAGTCTCCGAGGC |
|  | *HK2-201(CA)* | TCGCCTGCTTATTCACGGAG | CATCTGAGAGACGCATGTGGT |
|  | *Hkdc1-201 (CA)* | CACCAAGCTGAAGGAGGACC | GGCCATGATGTCCACCAG |
|  | *Insig1-201 (CA)* | GTCACGCTCTTCCCCGAC | CAGTAAACCGACAACAGCCG |
|  | *Irs2-201 (CA)* | TGCAAGCATCGACTTCCTGT | TCAACATGGCGGCGATGG |
|  | *Jund1-201 (CA)* | GGGATTGAAACCAGGGTCGG | CGAGTTTGAGCAGCCCAAGA |
|  | *Ldha-210 (CA)* | AAGTCCTCAGGCGGCTACA | ACAATCAGCTGGTCCTTGAGG |
|  | *Maff-201 (CA)* | GATCCCTTATCTAGCAAAGCCCT | CTTCAGCCGCGTCACCTC |
|  | *Map3k14-201 (CA)* | ACGTGATCACCAAAGGCACA | GAGAAGGTGGGGCTGAACTC |
|  | *Mapkap2-201 (CA)* | TGCTGCGGATCTTCGACAAG | GTGCAGCTCCACCTCTCTG |
|  | *Mpi* | GGCCCAGATTTCAGAGGACA | CGGTTGTCAAGGATCTTGGC |
|  | *Nppb-201 (CA)* | TCCTAGCCAGTCTCCAGAGC | GTGCTGCCTTGAGACCGAA |
|  | *Pdha2-201 (CA)* | AATGTGACCTGTACCGGCTG | CCTGCATGGTCCGGTAGTAC |
|  | *Pfkfb1* | TCTCTACGAAGCTCACACGC | CTCACTGCCTCTCGTCGATAC |
|  | *Pfkfb2-206 (NMD)* | CCCCTGCACATCATCTTCAAAC | TGGCTCTTGGGGAAGTTGTG |
|  | *Pfkfb3-220 (CA)* | CCAGAGCCGGGTACAGAAGA | GAGCCCCACCATCACAATCA |
|  | *Pfkfb4-206 (RI)* | CAGCGTGGTGTCTGCATGA | AATCCAGTTGAGGTACCGCG |
|  | *PfkL-207 (PT)* | AAGGCCATTGGAGTGCTGAC | ATGAGGAAGACTTTGGCCCC |
|  | *PfkM-201 (CA)* | AGTTGGTATCTTCACGGGCG | CATAGACACGCTCTCCCACG |
|  | *PfkP-210 (CA)* | CGGTTGTGATACTGCCCTGA | AGCCACAGTATCCACCCATG |
|  | *Pgls-202 (RI)* | GAGCACGTACGGCCTCTAC | CCACAGGTAGGGCAGGATTG |
|  | *Pgm2l1- 201 (NMD)* | CCAGCTAGACACGGCCATC | CCTTGTTCATCCCATTCCGC |
|  | *PkM-203 (RI)* | GCGTGGAGGCCTCTTATAAGT | ACATAGCGGACCTAGTTGCG |
|  | *PkM-205 (RI)* | GAGGTCCAGCATTCAGAGCC | AGGAGTCAGGTCAGCTGGAA |
|  | *PkM-206 (CM)* | CCCGCATTCTAGTCACGTCC | GCTGGGTCTGAATGAAGGCA |
|  | *Prprsap1lncRNA- 207 (PT)* | TCTGTCTGAGCTTGCGTTTCT | ACACACAGGTCCCTCTCAGA |
|  | *RassF1-201 (CA)* | GAAATGACCTGGAGCAGCAC | GGTGTCTCCCGCTCTACAG |
|  | *Rpe-202* | GTCCATCCTCAACAGCGACC | AAACTGACCTCCCTGAAGAG |
|  | *Srpk1-212 (RI)* | TGTGAGAATTGTCCTGCTGCT | CTCCGTGAGTGTCTGGTCCT |

**Supplementary Table 2.** Antibodies used in immunofluorescence and western blotting studies.

| **Antibody** | **Source** | **Code/dilution** | **Technique** |
| --- | --- | --- | --- |
| Nanog | Abcam | 80892, 1:200 | Immunofluorescence |
| Gata4 | Santa Cruz | SC1237, 1:200 | Immunofluorescence |
| Oct4 | Santa Cruz | SC5279, 1:200 | Immunofluorescence |
| Bim | Cell Signaling | 2819, 1:700 | Western blotting |
| Cleaved-caspase 3 (Asp175) | Cell Signaling | 9661, 1:1000 | Western blotting |
| Caspase 3 | Cell Signaling | 9662, 1:1000 | Western blotting |
| Phospho-AKT (Ser493) | Cell Signaling | 4060, 1:1000 | Western blotting |
| Phospho-ERK (Thr202/Tyr204) | Cell Signaling | 9101, 1:1000 | Western blotting |
| Phospho-JNK (Thr183/Tyr185) | Cell Signaling | 4668, 1:1000 | Western blotting |
| Phospho-p38 (Thr180/Tyr182) | Cell Signaling | 4631 1:1000 | Western blotting |
| Phospho-Stat3 (Tyr705) | Cell Signaling | 9131, 1:1000 | Western blotting |
| Total-AKT | Cell Signaling | 9272, 1:1000 | Western blotting |
| Total-ERK | Cell Signaling | 4695 1:1000 | Western blotting |
| Total-p38 | Cell Signaling | 9212, 1:1000 | Western blotting |
| Puma | In house | 1:500 | Western blotting |
| Stat3 | Cell Signaling | 9132, 1:1000 | Western blotting |
| α-tubulin | Cell Signaling | 2144, 1:1000 | Western blotting |
