# Supplementary material for: Maternal Undernutrition Induces Cell Signalling and Metabolic Dysfunction in Undifferentiated Mouse Embryonic Stem Cells
Source: Stem Cell Rev Rep. 2022 Dec 15;19(3):767–83. doi: 10.1007/s12015-022-10490-1 (PMC10070223; doi:10.1007/s12015-022-10490-1)
Supplement: Supplementary file 2 — Supplementary file2 (PPTM 7104 KB) comprising Supplementary Figures 1-6. [file 12015_2022_10490_MOESM2_ESM.pptm]

## Slide 1
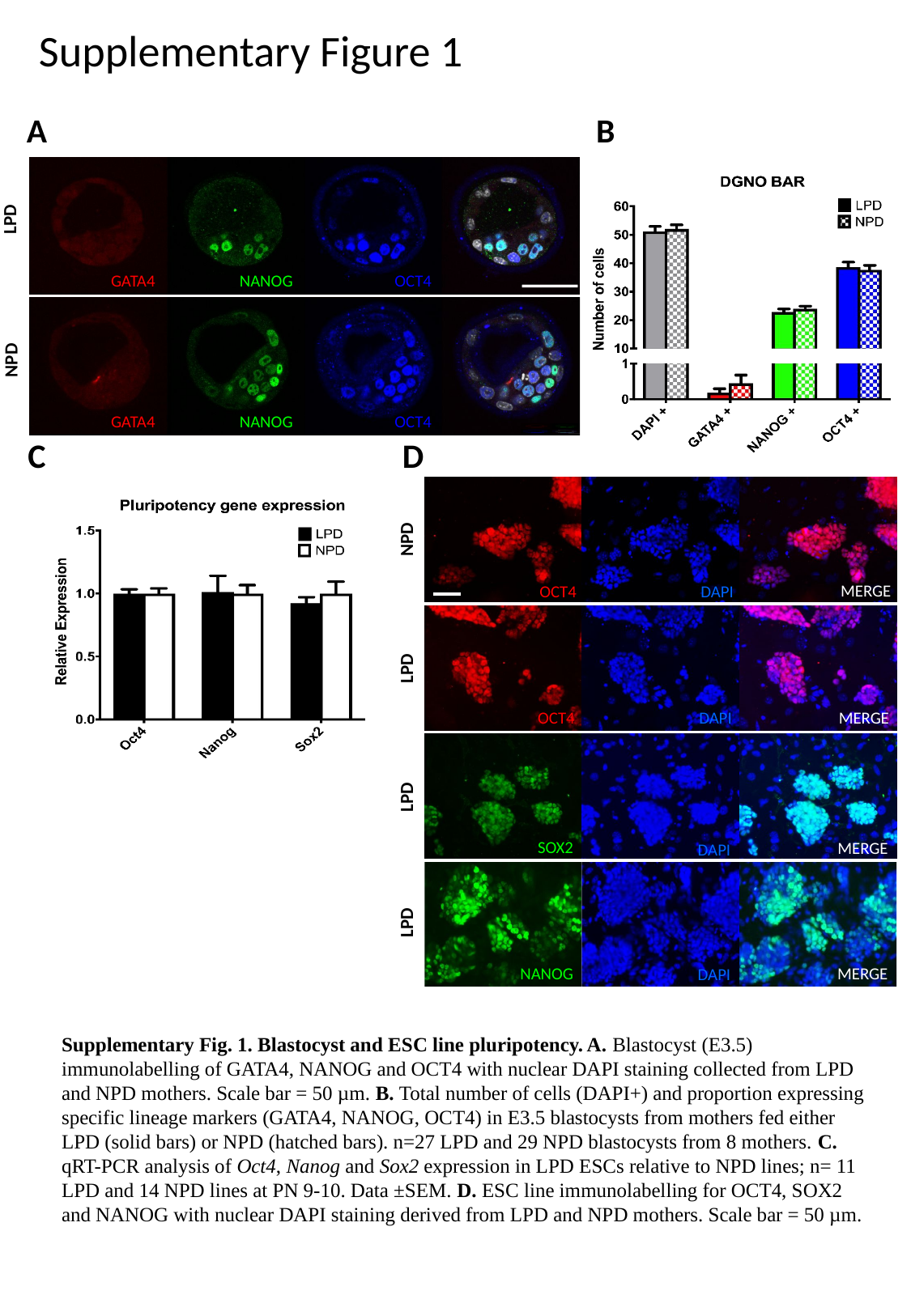

Supplementary Figure 1
A
B
LPD
GATA4
NANOG
OCT4
NPD
GATA4
NANOG
OCT4
C
D
NPD
MERGE
OCT4
DAPI
LPD
OCT4
DAPI
MERGE
LPD
SOX2
MERGE
DAPI
LPD
MERGE
NANOG
DAPI
Supplementary Fig. 1. Blastocyst and ESC line pluripotency. A. Blastocyst (E3.5) immunolabelling of GATA4, NANOG and OCT4 with nuclear DAPI staining collected from LPD and NPD mothers. Scale bar = 50 µm. B. Total number of cells (DAPI+) and proportion expressing specific lineage markers (GATA4, NANOG, OCT4) in E3.5 blastocysts from mothers fed either LPD (solid bars) or NPD (hatched bars). n=27 LPD and 29 NPD blastocysts from 8 mothers. C. qRT-PCR analysis of Oct4, Nanog and Sox2 expression in LPD ESCs relative to NPD lines; n= 11 LPD and 14 NPD lines at PN 9-10. Data ±SEM. D. ESC line immunolabelling for OCT4, SOX2 and NANOG with nuclear DAPI staining derived from LPD and NPD mothers. Scale bar = 50 µm.

## Slide 2
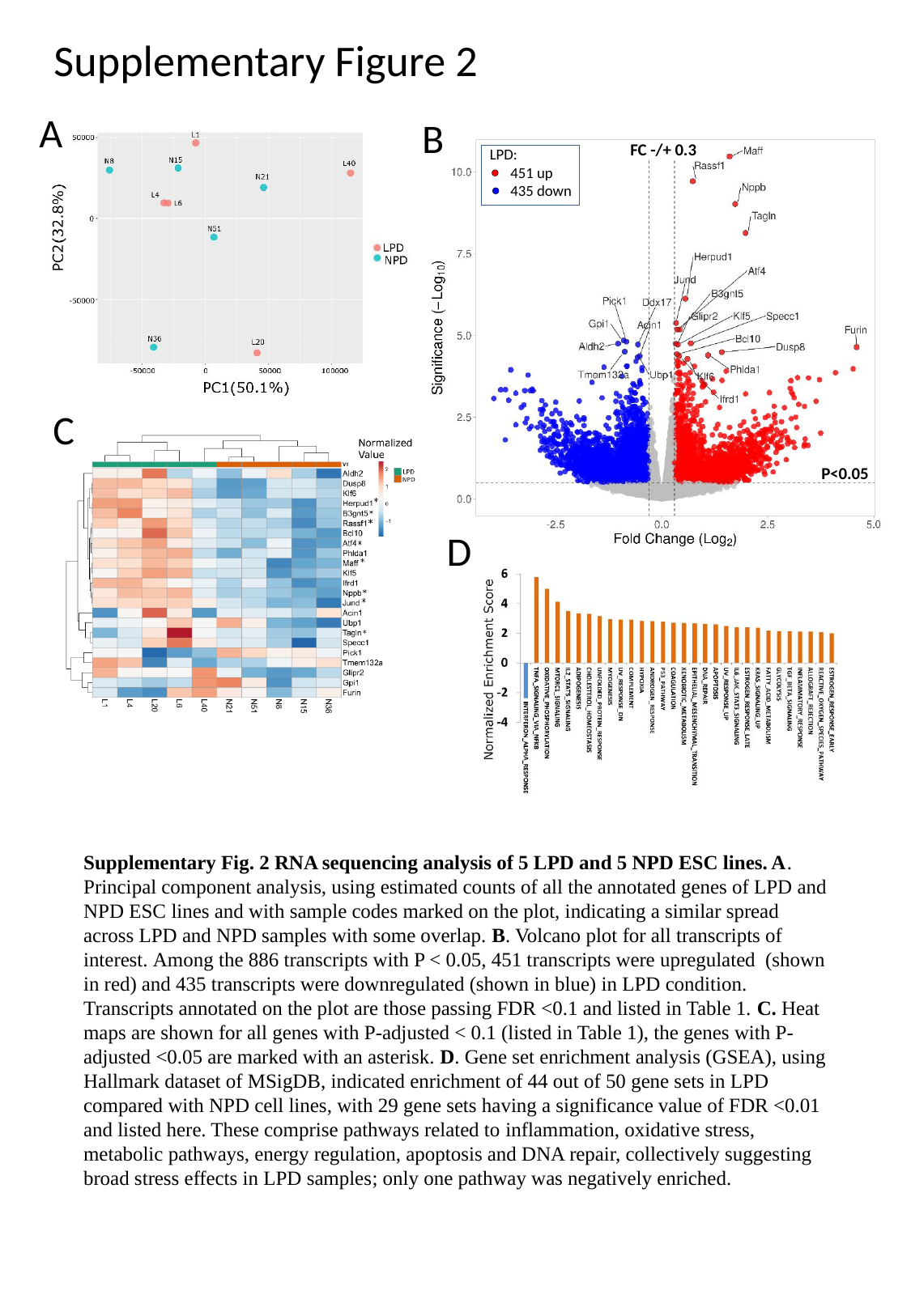

Supplementary Figure 2
A
B
FC -/+ 0.3
LPD:
 451 up
 435 down
P<0.05
C
D
Supplementary Fig. 2 RNA sequencing analysis of 5 LPD and 5 NPD ESC lines. A. Principal component analysis, using estimated counts of all the annotated genes of LPD and NPD ESC lines and with sample codes marked on the plot, indicating a similar spread across LPD and NPD samples with some overlap. B. Volcano plot for all transcripts of interest. Among the 886 transcripts with P < 0.05, 451 transcripts were upregulated (shown in red) and 435 transcripts were downregulated (shown in blue) in LPD condition. Transcripts annotated on the plot are those passing FDR <0.1 and listed in Table 1. C. Heat maps are shown for all genes with P-adjusted < 0.1 (listed in Table 1), the genes with P-adjusted <0.05 are marked with an asterisk. D. Gene set enrichment analysis (GSEA), using Hallmark dataset of MSigDB, indicated enrichment of 44 out of 50 gene sets in LPD compared with NPD cell lines, with 29 gene sets having a significance value of FDR <0.01 and listed here. These comprise pathways related to inflammation, oxidative stress, metabolic pathways, energy regulation, apoptosis and DNA repair, collectively suggesting broad stress effects in LPD samples; only one pathway was negatively enriched.

## Slide 3
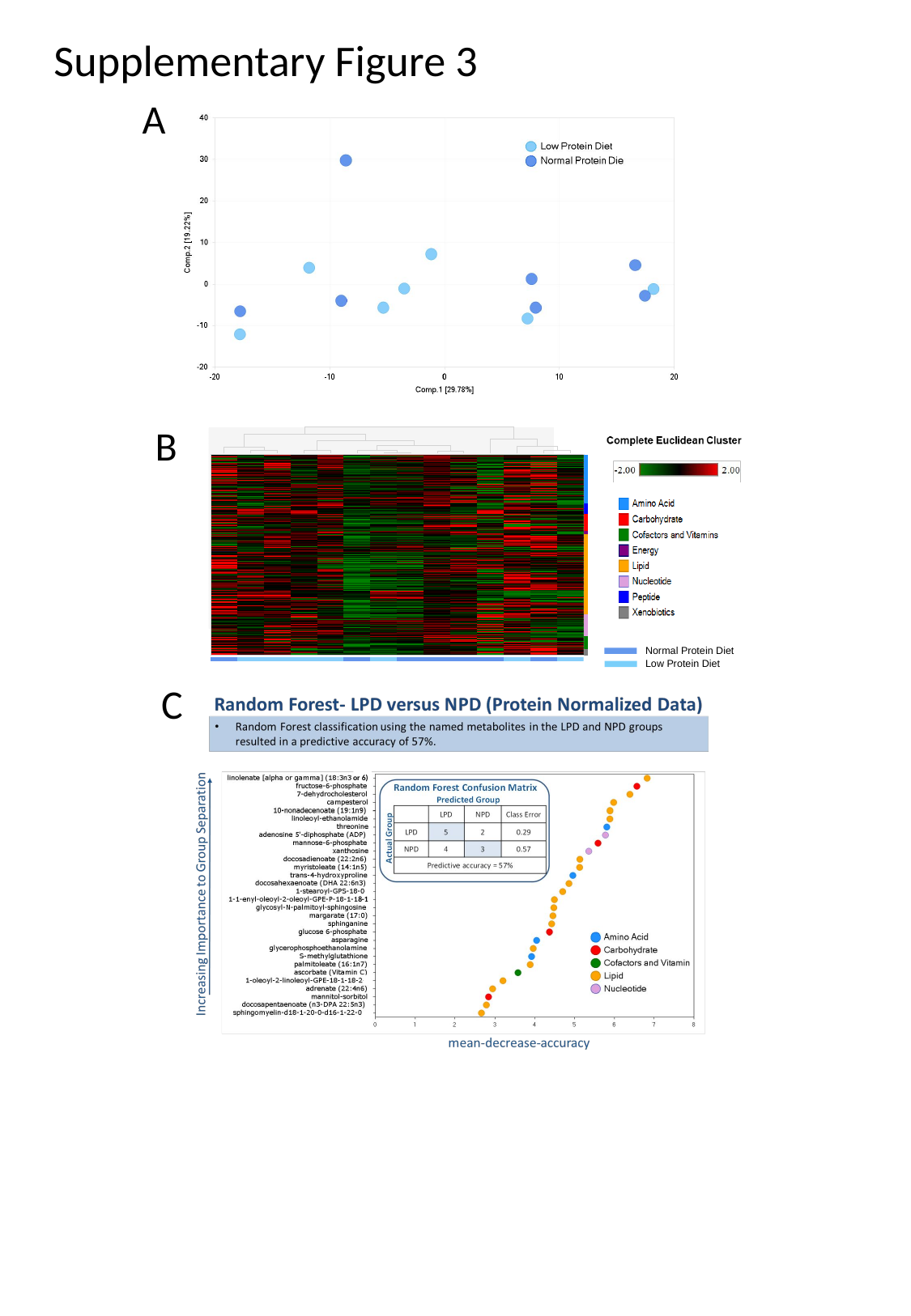

Supplementary Figure 3
A
B
Normal Protein Diet
Low Protein Diet
C
or 6)
8-1

## Slide 4
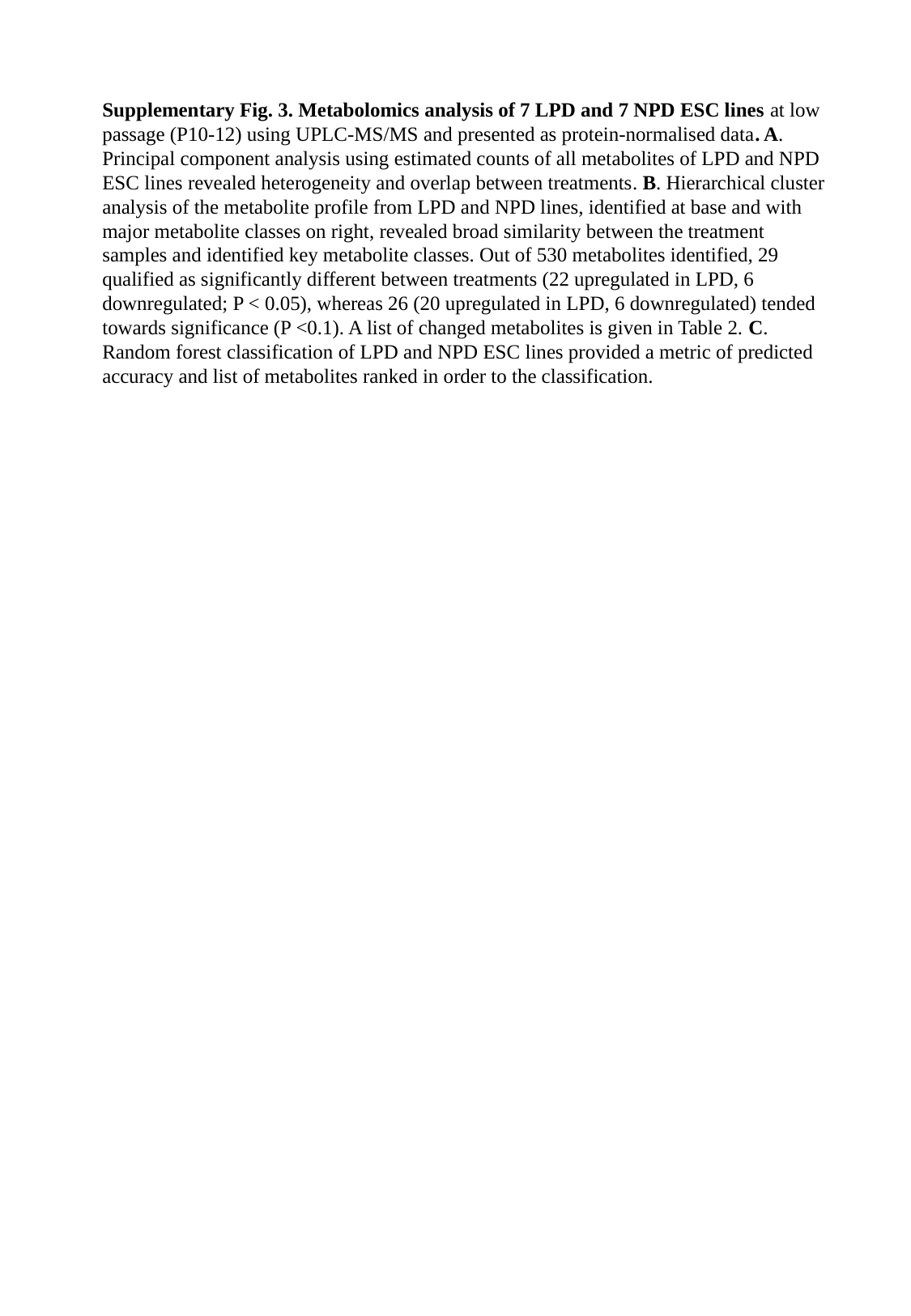

Supplementary Fig. 3. Metabolomics analysis of 7 LPD and 7 NPD ESC lines at low passage (P10-12) using UPLC-MS/MS and presented as protein-normalised data. A. Principal component analysis using estimated counts of all metabolites of LPD and NPD ESC lines revealed heterogeneity and overlap between treatments. B. Hierarchical cluster analysis of the metabolite profile from LPD and NPD lines, identified at base and with major metabolite classes on right, revealed broad similarity between the treatment samples and identified key metabolite classes. Out of 530 metabolites identified, 29 qualified as significantly different between treatments (22 upregulated in LPD, 6 downregulated; P < 0.05), whereas 26 (20 upregulated in LPD, 6 downregulated) tended towards significance (P <0.1). A list of changed metabolites is given in Table 2. C. Random forest classification of LPD and NPD ESC lines provided a metric of predicted accuracy and list of metabolites ranked in order to the classification.

## Slide 5
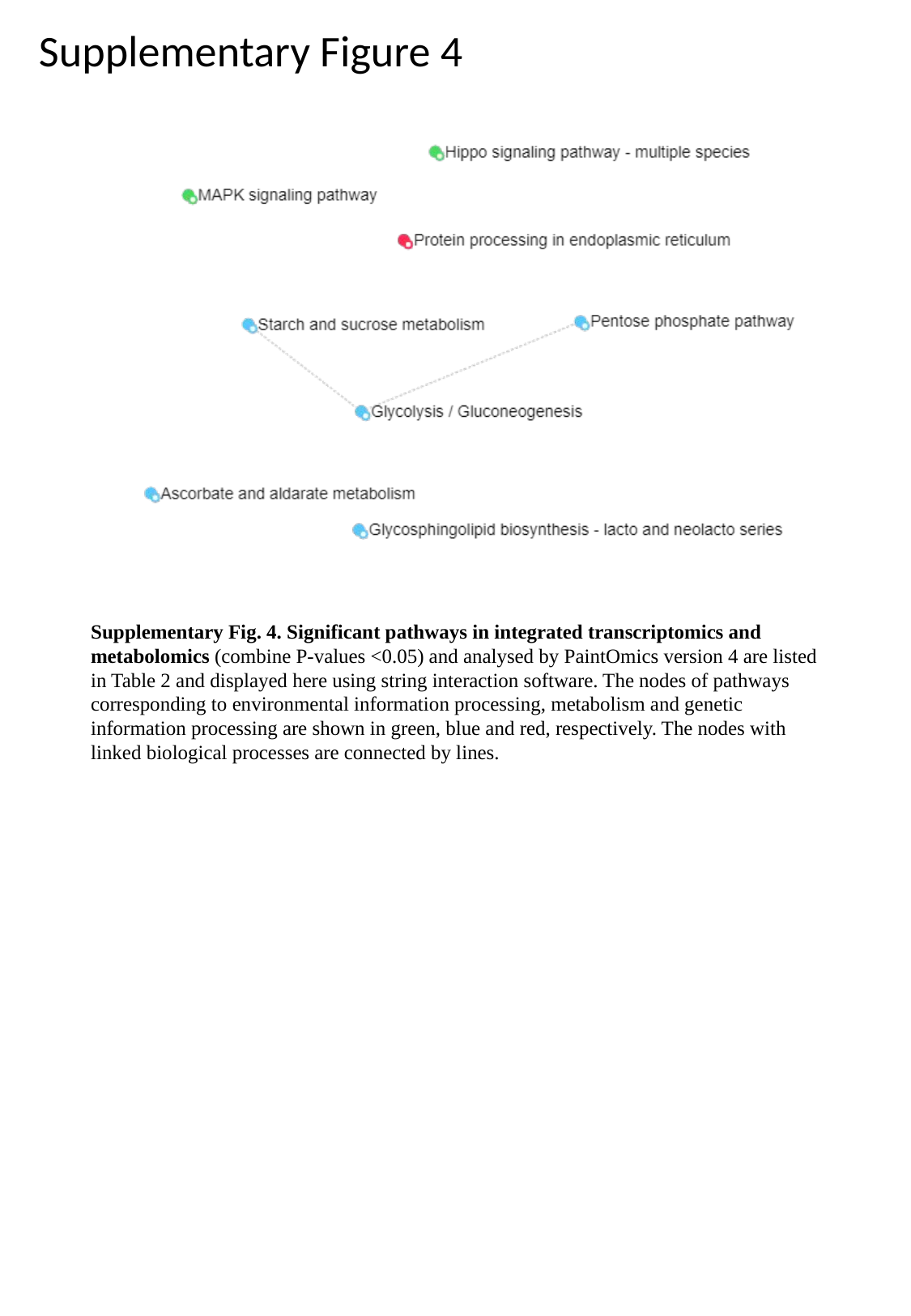

Supplementary Figure 4
Supplementary Fig. 4. Significant pathways in integrated transcriptomics and metabolomics (combine P-values <0.05) and analysed by PaintOmics version 4 are listed in Table 2 and displayed here using string interaction software. The nodes of pathways corresponding to environmental information processing, metabolism and genetic information processing are shown in green, blue and red, respectively. The nodes with linked biological processes are connected by lines.

## Slide 6
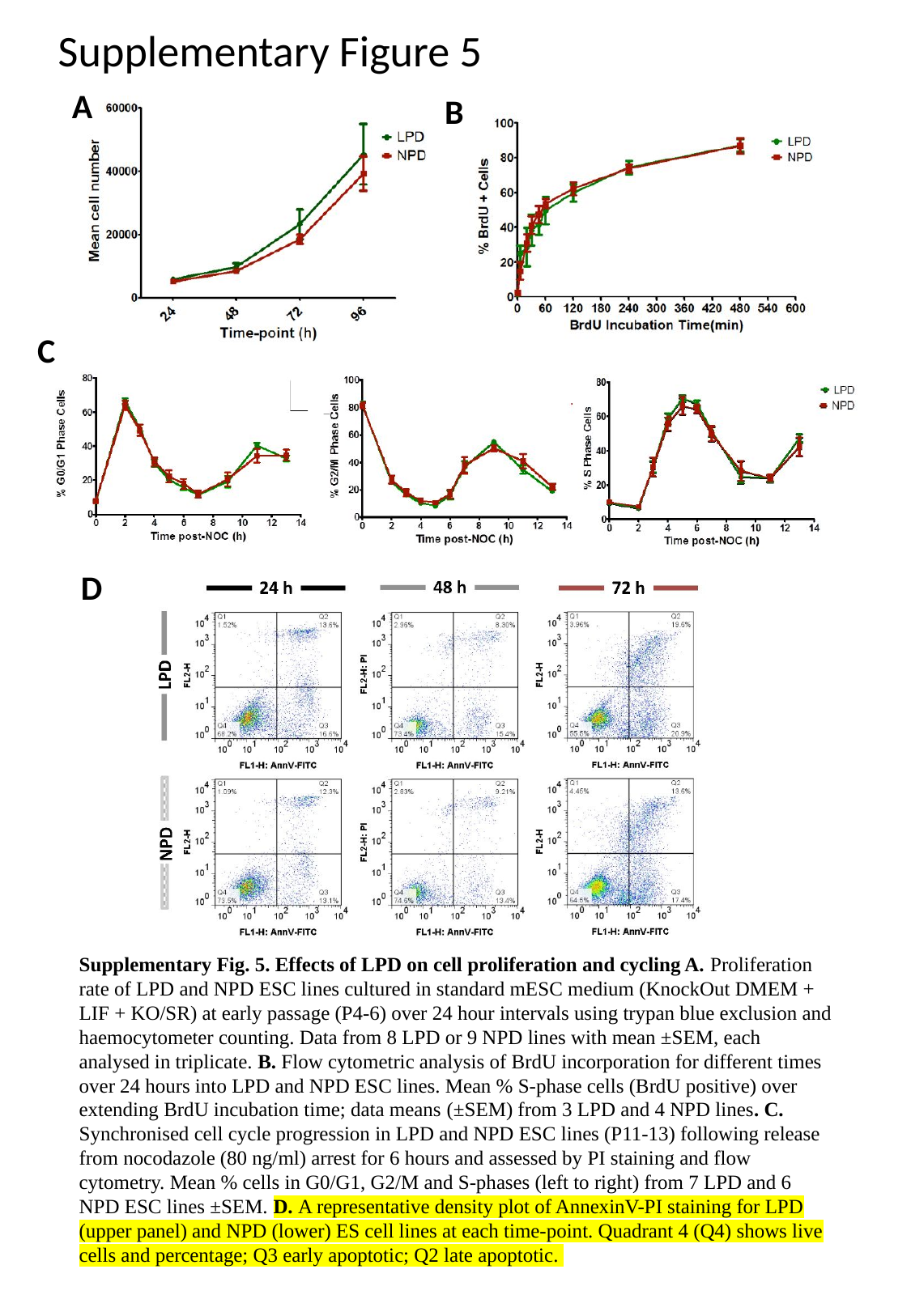

Supplementary Figure 5
A
B
C
D
Supplementary Fig. 5. Effects of LPD on cell proliferation and cycling A. Proliferation rate of LPD and NPD ESC lines cultured in standard mESC medium (KnockOut DMEM + LIF + KO/SR) at early passage (P4-6) over 24 hour intervals using trypan blue exclusion and haemocytometer counting. Data from 8 LPD or 9 NPD lines with mean ±SEM, each analysed in triplicate. B. Flow cytometric analysis of BrdU incorporation for different times over 24 hours into LPD and NPD ESC lines. Mean % S-phase cells (BrdU positive) over extending BrdU incubation time; data means (±SEM) from 3 LPD and 4 NPD lines. C. Synchronised cell cycle progression in LPD and NPD ESC lines (P11-13) following release from nocodazole (80 ng/ml) arrest for 6 hours and assessed by PI staining and flow cytometry. Mean % cells in G0/G1, G2/M and S-phases (left to right) from 7 LPD and 6 NPD ESC lines ±SEM. D. A representative density plot of AnnexinV-PI staining for LPD (upper panel) and NPD (lower) ES cell lines at each time-point. Quadrant 4 (Q4) shows live cells and percentage; Q3 early apoptotic; Q2 late apoptotic.

## Slide 7
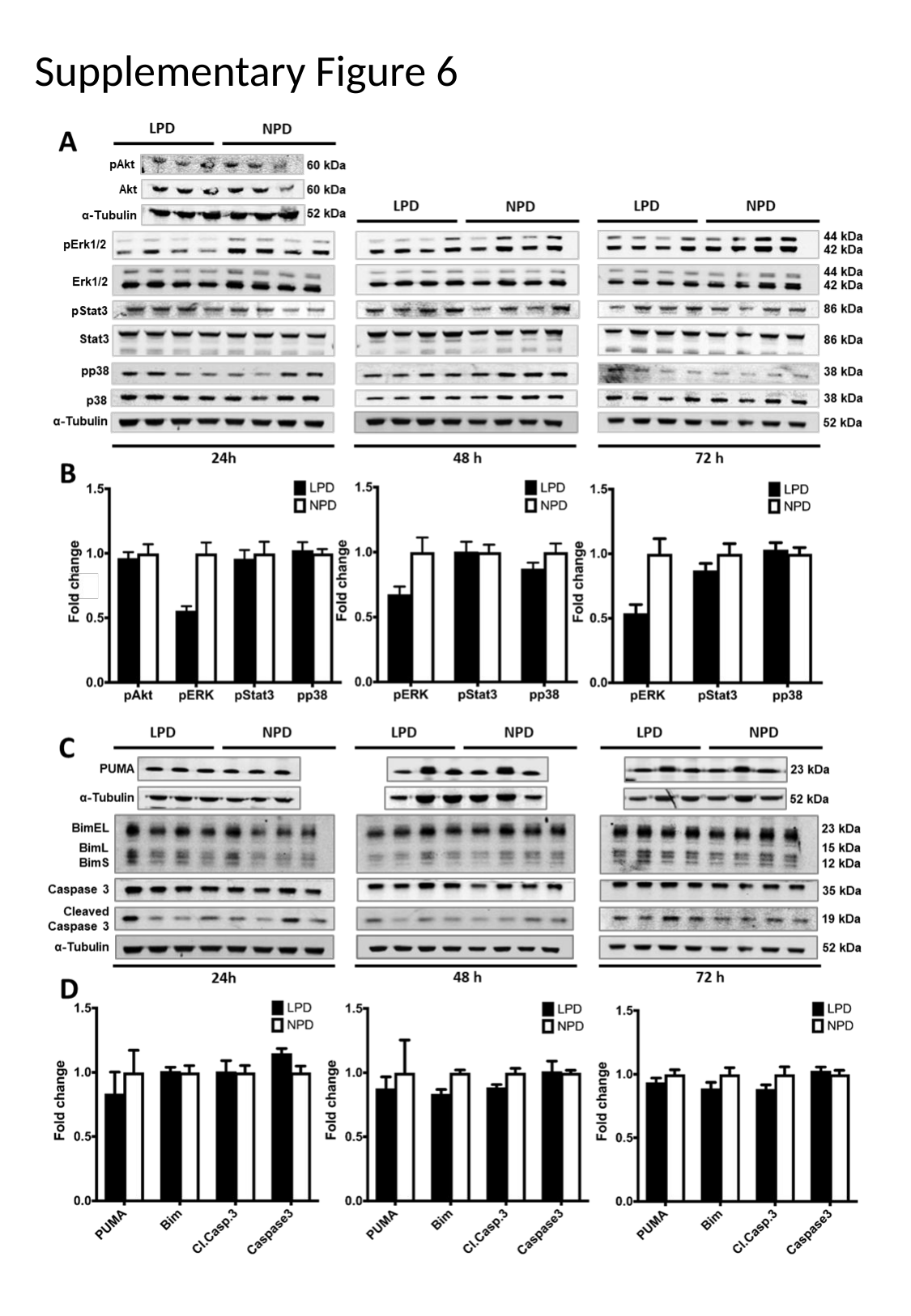

Supplementary Figure 6

## Slide 8
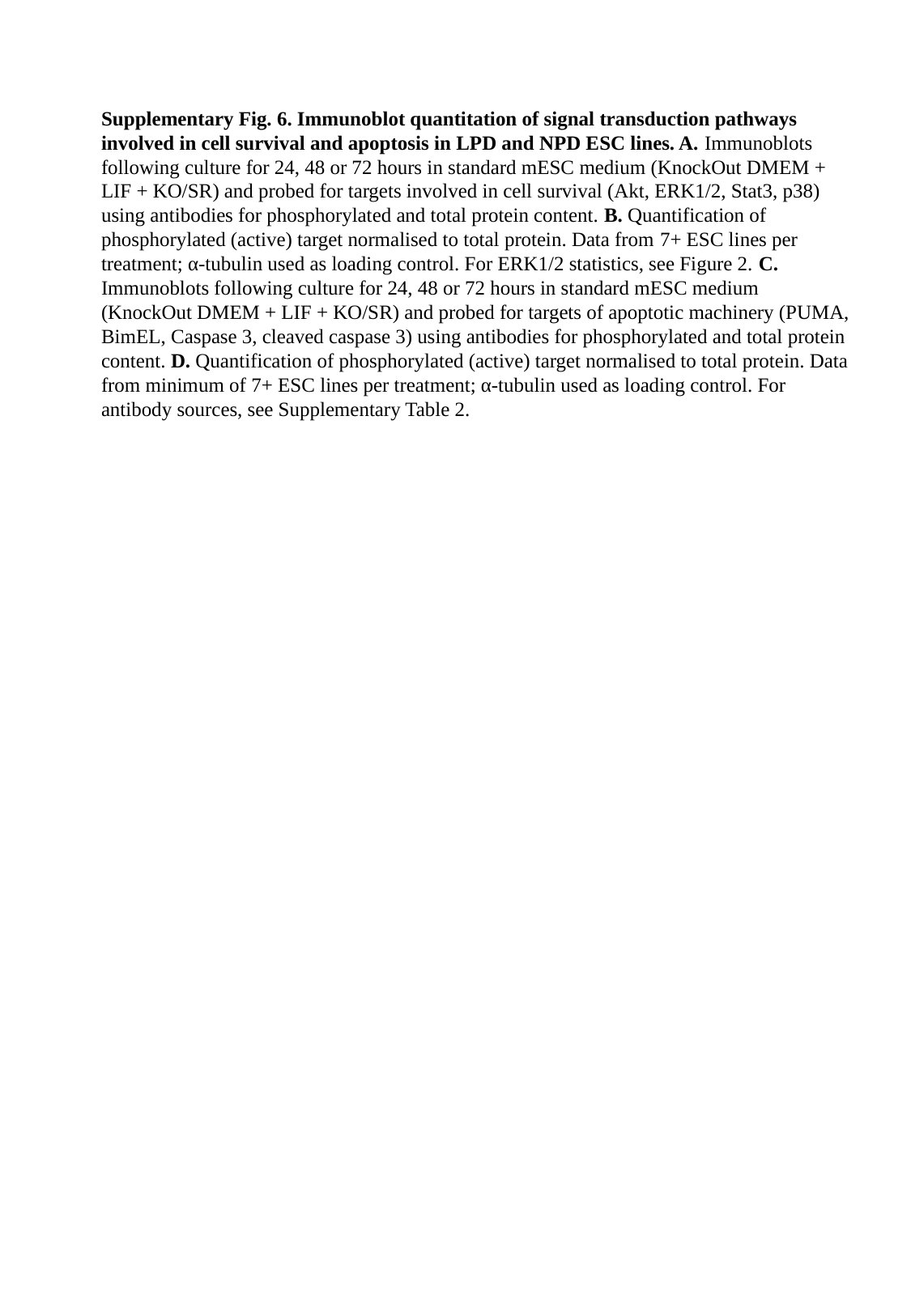

Supplementary Fig. 6. Immunoblot quantitation of signal transduction pathways involved in cell survival and apoptosis in LPD and NPD ESC lines. A. Immunoblots following culture for 24, 48 or 72 hours in standard mESC medium (KnockOut DMEM + LIF + KO/SR) and probed for targets involved in cell survival (Akt, ERK1/2, Stat3, p38) using antibodies for phosphorylated and total protein content. B. Quantification of phosphorylated (active) target normalised to total protein. Data from 7+ ESC lines per treatment; α-tubulin used as loading control. For ERK1/2 statistics, see Figure 2. C. Immunoblots following culture for 24, 48 or 72 hours in standard mESC medium (KnockOut DMEM + LIF + KO/SR) and probed for targets of apoptotic machinery (PUMA, BimEL, Caspase 3, cleaved caspase 3) using antibodies for phosphorylated and total protein content. D. Quantification of phosphorylated (active) target normalised to total protein. Data from minimum of 7+ ESC lines per treatment; α-tubulin used as loading control. For antibody sources, see Supplementary Table 2.
